# The Role of Intestinal Microbial Metabolites in the Immunity of Equine Animals Infected With Horse Botflies

**DOI:** 10.3389/fvets.2022.832062

**Published:** 2022-06-22

**Authors:** Dini Hu, Yujun Tang, Chen Wang, Yingjie Qi, Make Ente, Xuefeng Li, Dong Zhang, Kai Li, Hongjun Chu

**Affiliations:** ^1^Key Laboratory of Non-invasive Research Technology for Endangered Species, School of Ecology and Nature Conservation, Beijing Forestry University, Beijing, China; ^2^Xinjiang Research Centre for Breeding Przewalski's Horse, Ürümqi, China; ^3^Altay Management Station of Mt. Kalamaili Ungulate Nature Reserve, Altay, China; ^4^Institute of Forest Ecology, Xinjiang Academy of Forestry, Ürümqi, China

**Keywords:** *Equus przewalskii*, horse botfly, RNA sequencing, immune response, differentially expressed genes

## Abstract

The microbiota and its metabolites play an important role in regulating the host metabolism and immunity. However, the underlying mechanism is still not well studied. Thus, we conducted the LC-MS/MS analysis and RNA-seq analysis on *Equus przewalskii* with and without horse botfly infestation to determine the metabolites produced by intestinal microbiota in feces and differentially expressed genes (DEGs) related to the immune response in blood and attempted to link them together. The results showed that parasite infection could change the composition of microbial metabolites. These identified metabolites could be divided into six categories, including compounds with biological roles, bioactive peptides, endocrine-disrupting compounds, pesticides, phytochemical compounds, and lipids. The three pathways involving most metabolites were lipid metabolism, amino acid metabolism, and biosynthesis of other secondary metabolites. The significant differences between the host with and without parasites were shown in 31 metabolites with known functions, which were related to physiological activities of the host. For the gene analysis, we found that parasite infection could alarm the host immune response. The gene of “cathepsin W” involved in innate and adaptive immune responses was upregulated. The two genes of the following functions were downregulated: “protein S100-A8” and “protein S100-A9-like isoform X2” involved in chemokine and cytokine production, the toll-like receptor signaling pathway, and immune and inflammatory responses. GO and KEGG analyses showed that immune-related functions of defense response and Th17 cell differentiation had significant differences between the host with and without parasites, respectively. Last, the relationship between metabolites and genes was determined in this study. The purine metabolism and pyrimidine metabolism contained the most altered metabolites and DEGs, which mainly influenced the conversion of ATP, ADP, AMP, GTP, GMP, GDP, UTP, UDP, UMP, dTTP, dTDP, dTMP, and RNA. Thus, it could be concluded that parasitic infection can change the intestinal microbial metabolic activity and enhance immune response of the host through the pathway of purine and pyrimidine metabolism. This results will be a valuable contribution to understanding the bidirectional association of the parasite, intestinal microbiota, and host.

## Introduction

Horse botfly species are members of the *Gasterophilus* genus. These parasites live primarily in the gastrointestinal tract of equine animals but sometimes infect pigs, dogs, birds, and humans (1, 2). It has been reported that the prevalence of the horse botfly is particularly high in the desert steppe of Xinjiang, China. It infects nearly 100% of the wild equine animals that live there, which include *Equus przewalskii, Equus caballus*, and *Equus hemionus* (3). Due to horse botfly infestation, manifested as dysphagia, gastric and intestinal ulceration, and gastric obstruction or volvulus, gastrointestinal illnesses in equine animals can lead to various complications such as anemia, diarrhea, gastric rupture, peritonitis, and perforated ulcers (2, 4). When the parasite infects its host, it produces specific antigens during different stages in its life cycle, which induce the host to produce specific immune responses (5, 6). Thus, horse botfly infestation affects the physiology and immunity of equine animals.

Intestinal microbiota as an immune modulator plays an important role in maintaining the health of the host, which has extraordinary effect on physiology, metabolism, and immune function (7, 8). The balanced intestinal microbiota requires high microbial taxa diversity, high gene richness, and stable microbiome function (9). However, external factors such as age, diet, gender, and parasite infestation can disrupt the balance of intestinal microbiota, which results in the development of dysbiosis (10–12). The precise causes of the interaction between the unbalanced intestinal microbial community and the host health are that the metabolites produced by these microbes regulate the function of immune cells (13). For instance, the short-chain fatty acids (SCFAs) of butyrate derived from microbiota can promote IL-22 production to maintain the homeostasis of intestines (14, 15). Another SCFAs of lactate produced by *Bifidobacterium* spp. and lactic acid bacteria inhibit pro-inflammatory signals in the GPR81-independent metabolic process (16, 17). The metabolites derived from the microbiota profoundly shape the immunity, which means that understanding the microbial metabolic activities can help us clarify the immune status of the host.

Previous studies have shown that cytokine- or leukocyte-related gene expression in the blood can effectively reflect the host immune response in horses (18–22). But most of the studies on associations between parasite infections and immune responses in equine animals have mainly studied parasite antigen-induced host antibodies (23–25), and immune cells (26), clinical signs (27–30), and pathophysiology (31) in the host. It is our knowledge that only one study has evaluated gene expression in the whole blood of domestic horses infected with cyathostomin parasites (32). In addition, the study of the interaction between parasite infestation and intestinal microbiota in equine animals is still in its infancy. Starting in 2018, Peachey et al. (33) provided a novel insight into the parasite–microbiota interaction in equids. Following that, two studies explored these interactions in 2019 Walshe et al. (34) and Peachey et al. (35). At present, only Walshe et al. (34) examined the blood samples in horses infected with cyathostomin, who observed that the changes in the intestinal microbial community might be involved in increasing inflammatory markers like albumin and serum fibrinogen. Peachey et al. (35) identified different types of fecal metabolites when the parasite counts are high and low. However, so far, although Peachey et al. found that parasite infection can increase the metabolites of isobutyrate, trehalose, leucine, phenylalanine, glutamate, glucose, lysine, and propionate from microbiota and decrease metabolites of nicotinate, valerate, and butyrate, this study was conducted on equine youngstock that displayed a different microbial community pattern from adult animals (36). It is not known whether or not the immune genes are expressed in blood of infected horses. Therefore, there is still a lack of systematic study to clarify the role of intestinal microbiota in response to parasite infection and how the unbalanced microbiota affects the immunity of equine animals.

The previous study by our research group has determined that the horse botfly infestation on *E. przewalskii* can change some specific bacterial taxa (37) and also altered the archaea and eukaryotic and viral communities (38). In addition, the functional analysis showed that the intestinal microbiota mainly participated in the amino acid and carbohydrate metabolism in infected *E. przewalskii* (38). Our study will continuously investigate the associations between horse botfly infestation and intestinal microbiota of *E. przewalskii* through the identification of metabolites derived from microbiota in feces and immunity-related genes in blood, and link its effects on the metabolism and immunity more concretely. The results can provide further insights into the function of intestinal microbiota and host immune response of equine animals during parasite infection.

## Materials and Methods

### Ethics Statement

This study was conducted in accordance with Chinese law and the regulations of the Beijing Forestry University and its guidelines on animal research. The experimental protocol was reviewed and approved by the Institution of Animal Care and the Ethics Committee of the Beijing Forestry University (EAWC_BJFU_2020012). The management authority of the Kalamaili Nature Reserve (KNR) in China's Xinjiang region approved the collection of feces, botfly larvae, and blood from wild *E. przewalskii*.

### Fecal Metabolite Analysis

#### Fecal Sample Collection

**Figure 1:**
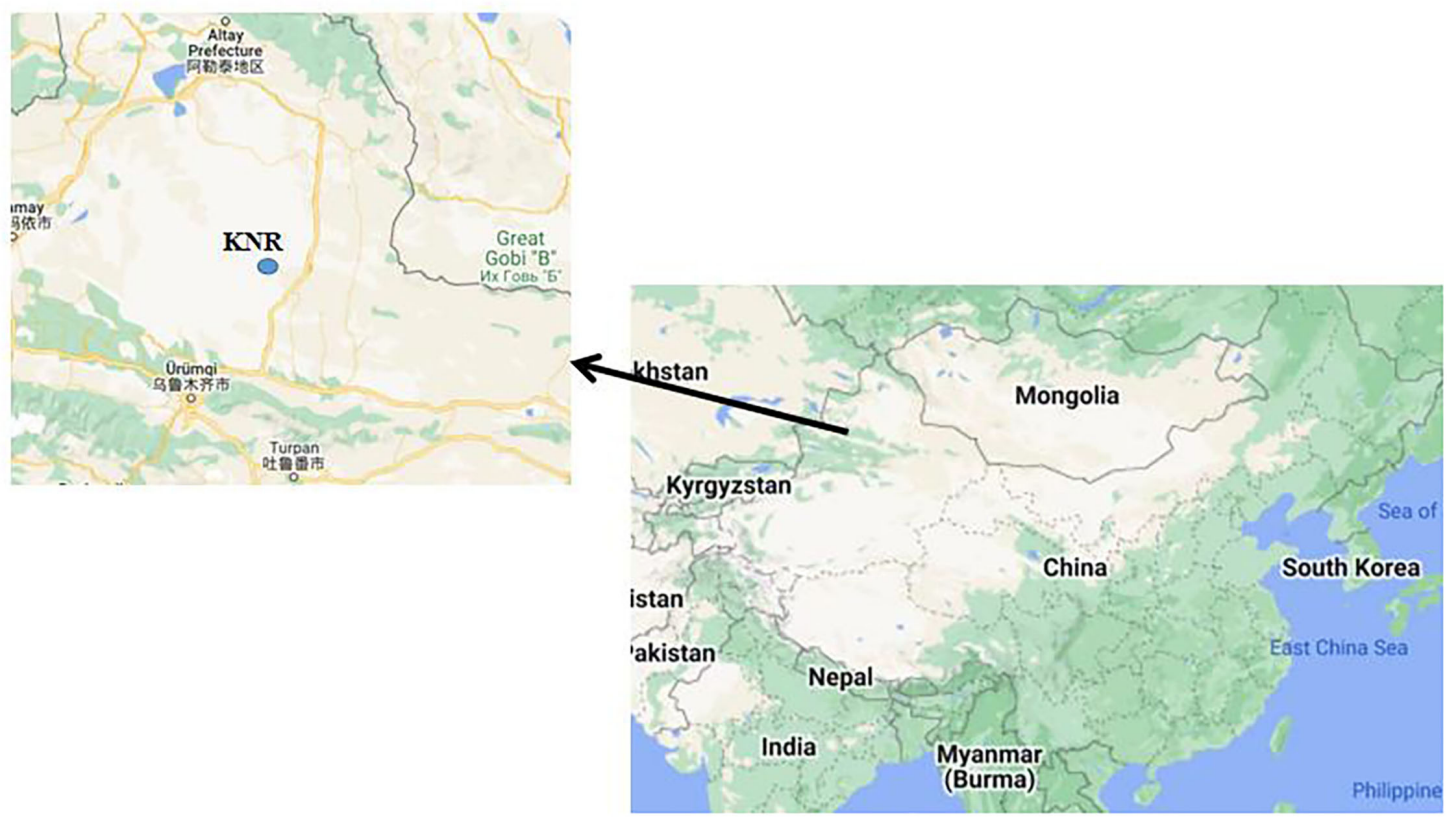
Location of KNR. The figure was generated by Google Maps. Arrow represents the location of Urumqi.

The procedures for fecal sample collection in Hu et al. (37) were adopted in our study. This study was carried out at the KNR in 2019 (Figure 1). In total, seven adult Przewalski's horses with similar body weight were driven from the wild and kept individually in a temporary enclosure. The fecal samples were collected from the horses immediately prior to the anthelmintic treatment (M-PATPHs) and 7 days thereafter (M-FATPHs). The fecal samples were stored in liquid nitrogen immediately after the collection and then transported back to the laboratory and stored at −80°C.

#### Metabolite Extraction and Quality Control Sample (QC) Preparation

A measure of 50 mg feces from each sample were used for chemical analysis. The solution of 400 μl methanol:water (4:1, v/v) was used for metabolite extraction. The mixture precipitated at −20°C was treated by using a high-throughput tissue crusher Wonbio-96c (Shanghai Wanbo Biotechnology Co., Ltd.) under the condition of 50 Hz for 6 min, followed by vortexing for 30 s, and ultrasound at 40 kHz and 5°C for 30 min. The prepared samples were placed at −20°C for 30 min for the precipitation of proteins, and the supernatant was centrifuged at 13,000 g at 4°C for 15 min and then carefully transferred to sample vials for liquid chromatography–mass spectrometry (LC-MS/MS) analysis. The QC was prepared by a mixture of equal volumes of all samples, followed by the same manner applied for analytic samples.

#### LC-MS/MS Analysis

A Thermo UHPLC system equipped with an ACQUITY BEH C18 column (100 mm × 2.1 mm i.d., 1.7 μm; Waters, Milford, USA) was used to perform the chromatographic separation of the metabolites. The mobile phases consisted of 0.1% formic acid in water (solvent A) and 0.1% formic acid in acetonitrile:isopropanol (1:1,v/v; solvent B). The solvent gradient was adjusted for equilibrating the systems according to the following conditions: 95% (A): 5% (B) to 80% (A): 20% (B), 0–3 min; 80% (A): 20% (B) to 5% (A): 95% (B), 3–9 min; 5% (A): 95% (B) to 5% (A): 95% (B), 9–13 min; 5% (A): 95% (B) to 95% (A): 5% (B), 13–13.1 min; 95% (A): 5% (B) to 95% (A): 5% (B), 13.1–16 min. The 2 μl samples were injected into the equipment with the flow rate at 0.4 ml/min and a column temperature at 40°C. All samples were stored at 4°C during the period of analysis.

The mass spectrometric data were collected based on a Thermo UHPLC-Q Exactive Mass Spectrometer equipped with an electrospray ionization (ESI) source operating in either positive or negative ion mode. The optimal conditions were set as follows: Aus gas heater temperature at 400°C, sheath gas flow rate at 40 psi, Aus gas flow rate at 30 psi, ion-spray voltage floating (ISVF) at −2,800 V in the negative mode and 3,500 V in the positive mode, and normalized collision energy at 20-40-60 V. The mode of data-dependent acquisition (DDA) was performed for data acquisition. The data were collected in the range of 70–1,050 *m*/*z*.

#### Data Preprocessing and Annotation

The Progenesis QI 2.3 (Nonlinear Dynamics, Waters, USA) was applied for detection of peak and alignment of raw data after LC-MS/MS analyses. A data matrix consisting of the retention time (RT), mass-to-charge ratio (*m*/*z*) values, and peak intensity can be obtained from the preprocessing analysis. The accurate mass, MS/MS fragments spectra, and isotope ratio difference were determined in comparison to the biochemical databases of Human Metabolome Database (HMDB; http://www.hmdb.ca/) and Metlin database (https://metlin.scripps.edu/), which were used for identification of metabolic features.

#### Multivariate Statistical and Differential Metabolite Analysis

The ropls (version 1.6.2, http://bioconductor.org/packages/release/bioc/html/ropls.html) R package from Bioconductor on Majorbio Cloud Platform (https://cloud.majorbio.com) was used for performing the multivariate statistical analysis. The differential metabolites between the comparable groups were determined through orthogonal partial least squares discriminate analysis (PLS-DA). Paired Student's *t*-test on single dimensional statistical analysis was adopted for estimating the *p-*values. The differential metabolites were selected based on the standard of *p*-value <0.05 and VIP value > 1. The database of Kyoto Encyclopedia of Genes and Genomes (KEGG, http://www.genome.jp/kegg/) was applied for the clarification of the participated biochemical pathways and the involved functions of the differential metabolites.

### Blood Transcriptome Analysis

#### Blood Sample Collections

Because no wild equine animals in the natural environment are free from parasites, the group of ivermectin-treated *E. przewalskii* was used as the control group (B-FATPHs). Lucja et al. (39) determined that immune cytokines were present at similar levels, and only four genes were differentially expressed in the blood when they compared children who were not infected with *Schistosoma haematobium* and children who had *S. haematobium* removed with praziquantel treatment 5 h after the treatment. Thus, in the present study, the groups of animals treated with the deworming drug are considered uninfected with *E. przewalskii* parasites.

With the veterinarian's assistance, blood samples from each horse immediately prior to the anthelmintic treatment (B-PATPHs) and 7 days thereafter (B-FATPHs) were collected through jugular vein puncture (37, 40). The whole blood drawn from each horse was placed in a blood collection tube (5 ml) and inverted several times. The blood samples were stored at −80°C until RNA was extracted.

#### RNA Extraction

Total RNA was extracted from the whole blood samples using TRIzol reagent in accordance with the manufacturer's instructions (Invitrogen, Carlsbard, CA, USA). DNase I (TaKara) was used to remove the genomic DNA. The 2100 Bioanalyzer (Agilent Technologies, Inc., Santa Clara CA, USA) and ND-2000 instrumentation (NanoDrop Thermo Scientific, Wilmington, DE, USA) were applied to determine the integrity and purity and quantify of the RNA. Only high-quality RNA samples (OD 260/280 = 1.8–2.2, OD 260/230 ≥ 2.0, RIN ≥8.0, 28S:18S ≥1.0, >1μg) were used to construct sequencing libraries.

#### Library Construction and Transcriptome Sequencing

RNA purification, reverse transcription, library construction, and sequencing were performed at Shanghai Majorbio Bio-pharm Biotechnology Co., Ltd. (Shanghai, China) in accordance with the manufacturer's instructions (Illumina, San Diego, CA). The Illumina TruSeqTM RNA sample preparation kit (Illumina) was used to prepare the whole-blood RNA-seq transcriptome libraries. The poly(A) mRNA purified from the total RNA using oligo-dT-attached magnetic beads was fragmented in a fragmentation buffer. Taking these short fragments as templates, double-stranded cDNA was synthesized using the SuperScript double-stranded cDNA synthesis kit (Invitrogen) with random hexamer primers (Illumina). The cDNA was synthesized after end-repair, and phosphorylation and “A” base addition were applied following the Illumina library construction protocol. Libraries were selected based on the size of cDNA fragments in the range of 200–300 bp on 2% Low Range Ultra Agarose, followed by PCR amplification using Phusion DNA polymerase (New England Biolabs, Boston, MA) for 15 PCR cycles. After fluorometric quantification on TBS380, two RNAseq libraries were sequenced in a single lane on the Illumina Hiseq xten/NovaSeq 6000 sequencer (Illumina) to obtain 2 × 150-bp paired-end reads.

#### *De novo* Assembly and Annotation

The default parameters of SeqPrep (https://github.com/jstjohn/SeqPrep) and Sickle (https://github.com/najoshi/sickle) were applied to trim the raw paired-end reads for quality control. The *de novo* assembly was performed using Trinity (http://trinityrnaseq.sourceforge.net/) (41). All the assembled transcripts were compared with the following databases: National Center for Biotechnology Information (nonredundant proteins, NR, https://www.ncbi.nlm.nih.gov/), Swiss-prot, Pfam, COG, and KEGG using BLASTX to identify the proteins with the highest sequence similarities to the given transcripts to retrieve their functional annotations using a typical cut-off *E*-value of ≤ 1.0 × 10^−5^. GO annotations of the unique assembled transcripts were obtained based on the BLAST2GO program to describe the biological processes, molecular functions, and cellular components (http://www.blast2go.com/b2ghome) (42). The KEGG database was employed to analyze the metabolic pathways (43).

#### Differential Expression Analysis and Functional Enrichment

To identify DEGs between two different samples, the expression level of each transcript was calculated in accordance with the transcripts per million reads (TPM) method. The gene abundances were quantified by RSEM (http://deweylab.biostat.wisc.edu/rsem/) (44). DESeq (45) and EdgeR (46) were used to perform the differential expression analysis. The *Q* values ≤ 0.05 and DEGs with |log_2_FC| >1 were considered to be significant DEGs. Functional enrichment analyses, including GO and KEGG, were performed to identify which DEGs were significantly enriched in GO terms and metabolic pathways at a Benjamini–Hochberg (BH) *p*-adjusted value of ≤ 0.05 compared with the whole transcriptome background. GOATOOLS (https://github.com/tanghaibao/Goatools) and KOBAS (http://bioinfo.org/kobas) (47) were employed to analyze the GO functional enrichment and KEGG pathway. The R software package was used to conduct the statistical analyses.

## Results and Discussions

### Detection of Metabolites in All Samples

The chemical analysis showed that a total of 5,913 peaks were observed in the positive ion mode, which were classified into 297 types of metabolites. Meanwhile, a total of 4,869 peaks and 215 metabolites were identified in the negative ion mode. The number of identified metabolites largely depend on the host species variation and the chemical analysis method (48). For the equine animal, a total of 28 metabolites in all fecal samples with high and low parasite burden were identified in a previous study (35), which might be resulted from the feces of youngstock sampled and proton nuclear magnetic resonance (1H-NMR) used. PLS-DA analysis revealed that the composition of the metabolites in *E. przewalskii* fecal samples prior to the treatment (M-PATPHs) and the metabolites in *E. przewalskii* fecal samples following the treatment (M-FATPHs) were different either in the positive mode or in the negative mode (Supplementary Figures S1, S2), which reflected that horse botfly infestation can impact the metabolic activities of intestinal microbiota in horses.

It is known that more than 50% metabolites detected in the feces are from the intestinal microbiota ((49)). The metabolites were annotated based on the KEGG compound classification, which were compounds with biological roles, bioactive peptides, endocrine-disrupting compounds, pesticides, phytochemical compounds, and lipids. Of those functions, the metabolites with biological roles were classified into eight types in the KEGG database, of which the three most abundant metabolites were amino acids, phospholipids, and nucleosides and bases (Supplementary Figure S3). As reported, the fecal metabolites of amino acids, nucleosides, and nucleic acid bases are also abundant in donkeys (48, 50). Amino acids produced by microbes can enhance the immune response of hosts (51). In phytochemical compounds, the most abundant metabolites were flavonoids and isoflavonoids, followed by fatty acids, monolignols, sesquiterpenoids, and triterpenoids (Supplementary Figure S4). Flavonoids are a class of secondary metabolites from plants which can be degraded by intestinal microbes (52). Thus, the flavonoids identified in this study should be from the food of horses. It has been reported that fatty acids and isoprenoids can be involved in many physiological and immune processes (53, 54). Isoflavonoids possess a wide range of pharmacological properties, such as antibacterial function (55), which can alter the symbiosis relationship among microbes.

These identified metabolites can be involved in the seven KEGG pathways, which were organismal systems, human diseases, environmental information processing, cellular processes, metabolism, genetic information processing, and drug development (Supplementary Figure S5). The three pathways with the largest number of metabolites were lipid metabolism, amino acid metabolism, and biosynthesis of other secondary metabolites, all of which were from the pathway of metabolism. These results can confirm the previous function analysis for intestinal microbiota of *E. przewalskii*, which suggested that metabolism is the most important function of the microbiota in the intestine, and the intestinal microbiota of horses mainly participate in the amino acid metabolism and carbohydrate metabolism (37). The identified metabolites in our study were also involved in the carbohydrate metabolism, which was not a dominant metabolism (**Figure 6**). In addition, the lipid metabolism is usually related to the synthesis of dietary lipids and the use of dietary nutrients (56, 57). The most identified metabolites produced by microbes through the lipid metabolism confirm our previous assumption that horse botfly infestation can influence the digestive ability of horses (37, 38).

### Fecal Metabolites Altered by Horse Botfly Infestation

The different analyses showed that a total of 2,477 metabolites had significant differences (*p* < 0.05) between M-PATPHs and M-FATPHs. Of these metabolites, 143 had been named and 31 had known functions (Supplementary Table S1 and Table 1). Except for l-carnitine, all other functional metabolites were mainly involved in the pathway of metabolism (Table 1). The horse botfly increased the abundance of adenosine 3′-monophosphate, aldosterone, DTMP, guanosine, hyocholic acid, inosine, l-carnitine, LPC (18:1), LPC (18:3), LysoPC (15:0), LysoPC (18:0), LysoPC [18:3 (6Z,9Z,12Z)], phosphocholine, 5-dehydroavenasterol, 2-aminoethylphosphonic acid, 16-hydroxy hexadecanoic acid, and 21-deoxycortisol and decreased the abundance of the rest of the metabolites in Table 1. Among those increased metabolites, adenosine 3'-monophosphate has the function of inhibiting protein synthesis (58). Aldosterone and 21-deoxycortisol are the important physiological stress indicators (59–61). Guanosine and its derivatives have potent inhibitory property against viruses (62, 63). Hyocholic acid and its derivatives are known for its resistance to type 2 diabetes, and it is commonly found in pigs, and its trace amount can be found in humans (64). It is first found in horses. Inosine plays an important role in RNA modulation, gene translation, and purine biosynthesis (65). L-Carnitine is usually linked to the lipid metabolism in animals (66). Phosphocholine is a precursor and also a degradation product of choline (vitamin) (67). 2-Aminoethylphosphonic acid has antibacterial function (68). The aforementioned results suggest that the horse botfly can participate in the metabolism of hosts and exert some beneficial effects on the hosts. This can be the reason why even horse botfly infestation is harmful to horses but not fatal.

**Table 1:** Functions of named 31 metabolites which showed significant differences between M-PATPHs and M-FATPHs.

**Compound name**	**Formula**	**KEGG pathway**
16-Hydroxy hexadecanoic acid	C_16_H_32_O_3_	Metabolism
21-Deoxycortisol	C_21_H_30_O_4_	Metabolism
2-Aminoethylphosphonic acid	C_2_H_8_NO_3_P	Metabolism; environmental information processing
2-Formaminobenzoylacetate	C_10_H_9_NO_4_	Metabolism
2-Indanone	C_9_H_8_O	Metabolism
4-(2-Aminophenyl)-2	C_10_H_9_NO_4_	Metabolism
4-Hydroxy-2-quinolone	C_9_H_7_NO_2_	Metabolism
5-Dehydroavenasterol	C_29_H_46_O	Metabolism
9(S)-HOTrE	C_18_H_30_O_3_	Metabolism
9-OxoODE	C_18_H_30_O_3_	Metabolism
Adenosine 3′-monophosphate	C_10_H_14_N_5_O_7_P	Metabolism
Aldosterone	C_21_H_28_O_5_	Metabolism; drug development; organismal systems
Atrolactic acid	C_9_H_10_O_3_	Metabolism
Cytosine	C_4_H_5_N_3_O	Metabolism
Dopaquinone	C_9_H_9_NO_4_	Metabolism
DTMP	C_10_H_15_N_2_O_8_P	Metabolism; human diseases
Guanosine	C_10_H_13_N_5_O_5_	Metabolism; environmental information processing
Hyocholic acid	C_24_H_40_O_5_	Metabolism
Inosine	C_10_H_12_N_4_O_5_	Metabolism; environmental information processing
l-Carnitine	C_7_H_15_NO_3_	Organismal systems
LPC(18:1)	C_26_H_52_NO_7_P	Metabolism; human diseases
LPC(18:3)	C_26_H_48_NO_7_P	Metabolism; human diseases
l-Proline	C_5_H_9_NO_2_	Metabolism; organismal systems; environmental information processing; genetic information processing; human diseases
LysoPC(15:0)	C_23_H_48_NO_7_P	Metabolism; human diseases
LysoPC(18:0)	C_26_H_54_NO_7_P	Metabolism; human diseases
LysoPC(18:3(6Z,9Z,12Z))	C_26_H_48_NO_7_P	Metabolism; human diseases
ParaXanthine	C_7_H_8_N_4_O_2_	Metabolism
Phosphocholine	C_5_H_14_NO_4_P	Metabolism; human diseases
Phytosphingosine	C_18_H_39_NO_3_	Metabolism
Stercobilin	C_33_H_46_N_4_O_6_	Metabolism
Xanthurenic acid	C_10_H_7_NO_4_	Metabolism

### Comparison of Total Gene Expression Between Horses With and Without Horse Botfly Infestation

**Figure 2:**
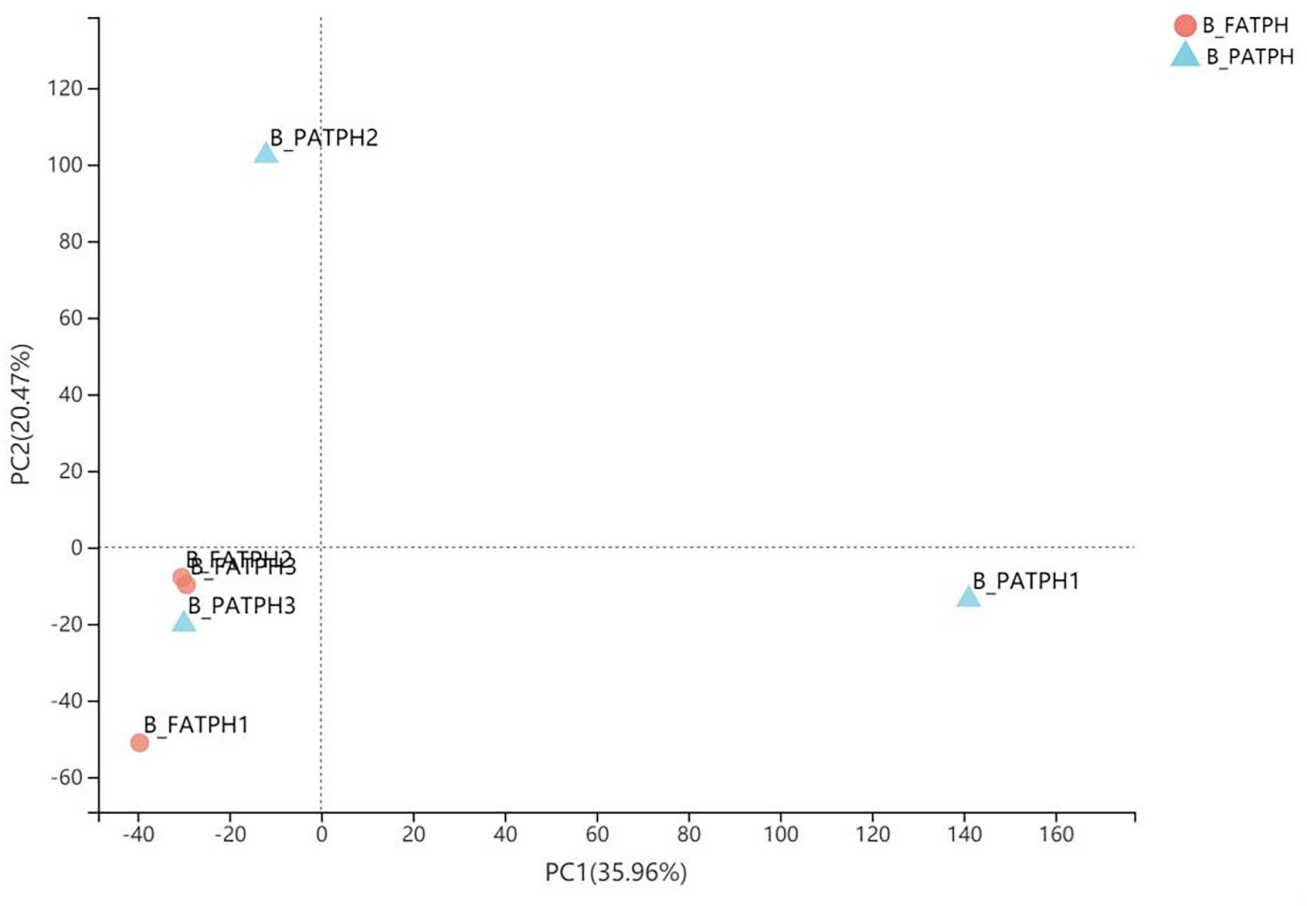
PCA plot showing the differences in the total number of DEGs between B-PATPHs (blue) and B-FATPHs (red) based on RSEM software analysis and the TPM index.

Only the RNA of the blood samples from three Przewalski's horses prior to and following the ivermectin treatment was of sufficient quality for library construction and transcriptome analysis. Altogether, 38.85 GB of clean data with Q30 percentages above 92.26% were obtained from the transcriptome analysis of the six samples (three horses, before and after treatment). Trinity was used to assemble the clean data, which identified 146,447 unigenes and 176,623 transcripts, and the average N50 length was 1,580 bp. The mapping rates ranged from 83.67 to 86.75% after comparing the clean reads from each sample with the reference sequences obtained from the Trinity assembly. In total, 1,564 DEGs were identified, of which 354 were upregulated and 1,210 were downregulated when comparing *E. przewalskii* blood samples prior to the treatment (B-PATPHs) with *E. przewalskii* blood samples following the treatment (B-FATPHs). The unigene annotation of six databases, including NR, Swiss-prot, Pfam, COG, GO, and KEGG, was used to determine functional information about the up- and downregulated genes. The analysis showed that most of the unigenes were annotated in NR (35,041, 24.90%), Swiss-prot (24,855, 17.67%), and COG (24,312, 17.28%). Among the unigenes, 37,954 successfully annotated in seven databases, accounting for 26.98%. The principal component analysis (PCA), which is used to visualize the expression differences and similarities between the B-PATPHs and B-FATPHs, showed that the whole-blood transcriptome profiles of the B-FATPHs clustered together, but the B-PATPHs were separated from each other, with 35.96 and 20.47% of the variation attributed to PC1 and PC2, respectively (Figure 2).

### Identifying DEGs Between Horses With and Without Horse Botfly Infestation and Analyzing Functional Enrichment

**Figure 3:**
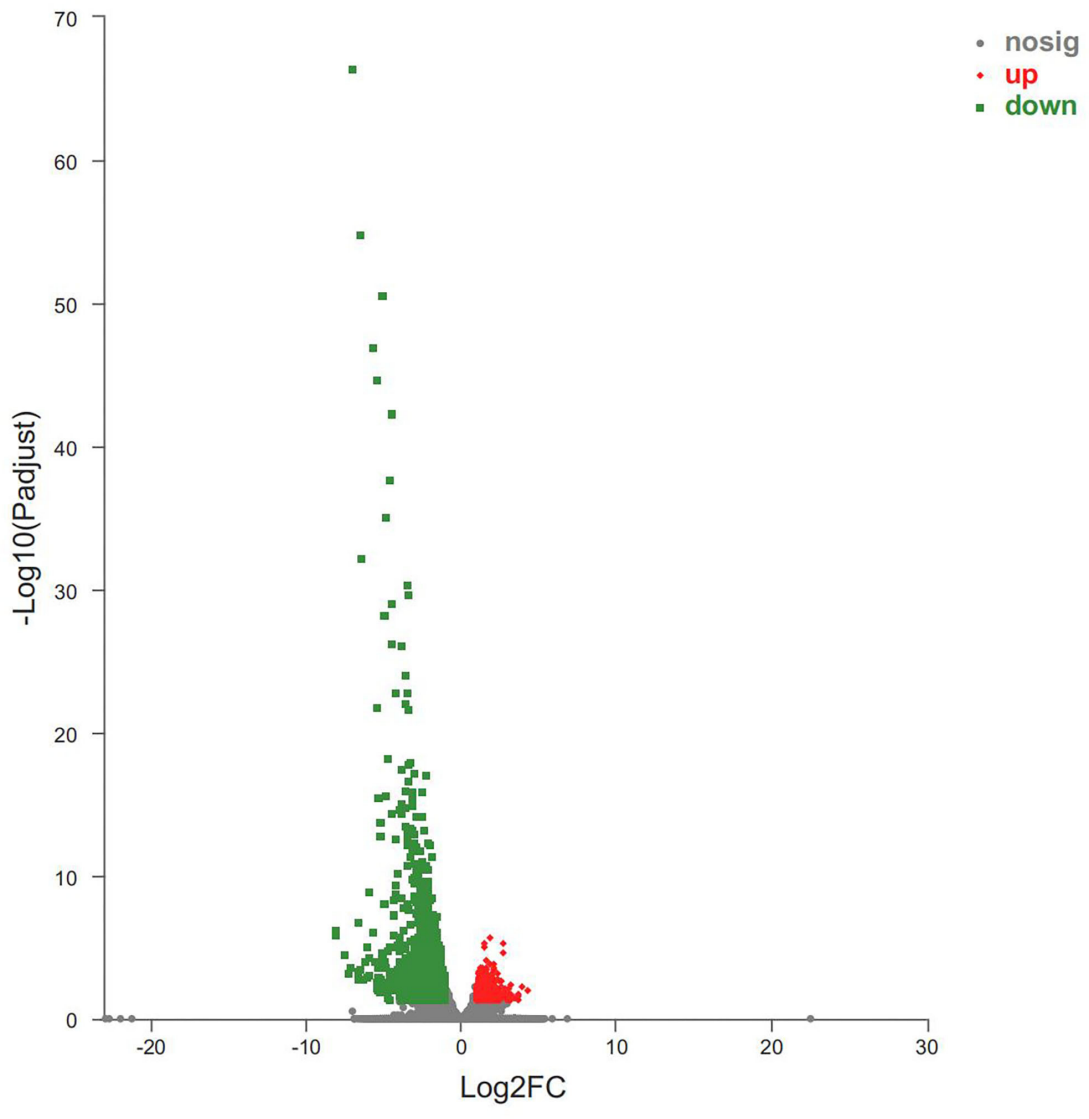
Volcano plot of DEGs between B-PATPHs and B-FATPHs. The *x*-axis indicates the fold-change, and the *y*-axis shows the statistically significant differential expression. The red, green, and gray dots represent upregulated unigenes, downregulated unigenes, and the unigenes with no significant changes, respectively.

Host responses to the botfly infestations were investigated by identifying the top DEGs between the B-PATPHs and B-FATPHs. The DEGs were considered to have a significant difference between the groups when the log_2_FC values exceeded 2, and the BH *p-*adjusted values were below 0.05. The volcano plot shown in Figure 3 indicates that the top upregulated DEGs included “protein FAM19A3 isoform X2” (source: *E. caballus, p* = 2.47E-06), “RNA binding motif” (*p* = 5.00E-06), and “cathepsin W” (source: *E. caballus, p* = 9.89E-06), of which cathepsin W is involved in proteolysis and cellular protein catabolic process (GO:0051603), extracellular space (GO:0005615), lysosome pathway (GO:0005764), and cysteine-type endopeptidase activity (GO:0004197; GO:0008234). The protein product of the cathepsin W gene also participates in apoptosis and lysosome pathways (K08569). Previous studies have also shown that the cathepsin gene is involved in the development of innate and adaptive immune responses of mammalian hosts during infection with helminth parasites (69, 70). Apoptosis is an immune-related pathway involved in the genetic programming process used by multicellular organisms to regulate their cell numbers or to eliminate harmful cells (71).

Meanwhile, the top downregulated DEGs were also identified in this study. They were “protein S100-A12” (source: *Tupaia chinensis, p* = 4.93E-67), “protein S100-A8” (source: *E. przewalskii, p* = 1.89E-55), “PREDICTED: protein S100-A9-like isoform X2” (source: *E. przewalskii, p* = 1.24E-47), “neutrophil gelatinase-associated lipocalin isoform X1” (source: *E. caballus, p* = 6.14E-43), “NADPH: adrenodoxin oxidoreductase, mitochondrial isoform X4” (source: *E. caballus, p* = 2.34E-38), “histidine ammonia-lyase” (source: *Panthera tigris altaica, p* = 8.76E-36), and “cystatin-A” (source: *E. caballus, p* = 7.83E-33). Two of the downregulated genes are associated with immune functions (e.g., “protein S100-A8” and “protein S100-A9-like isoform X2”). Protein S100-A8 is associated with chemokine production (GO:0032602), cytokine production (GO:0001816), toll-like receptor signaling pathway (GO:0002224; GO:0035662), and the immune and inflammatory response (GO:0002523; GO:0002526; GO:0002544; GO:0045087; GO:0050727; GO:0050729; GO:0006954). The gene encoding S100-A9-like isoform X2 functions in immune and inflammatory responses (GO:0045087 and GO:0050727) and toll-like receptor 4 binding (GO:0035662). As reported previously, cytokines are key mediators of immunity and participate in multiple biological processes in the host, such as defense against parasitic infections, tissue repair, allergic inflammation, and metabolic homeostasis (72, 73). Recent studies have suggested that mice infected with *Neospora caninum* parasites have higher levels of cytokines (mainly IFN-γ and IL-12) than uninfected controls (74), whereas mice infected with *Trypanosoma cruzi* have higher TNF-α and 1L-1β cytokine levels than uninfected controls (75). Chemokines, such as CXCL10, have also been shown to play important roles in the development of immunity, and their levels show a reduced trend in parasite-infected hosts (76). TLRs contribute a great deal to the immune response by activating the immune system through pathogen recognition (77), a finding applicable to equine animals (19, 21). One study concludes that TLR expression levels will decrease significantly after the host is infected with parasites, to avoid an excessive immune response against the parasites (78). Our results are consistent with those of that study, that is, the toll-like receptor signaling pathway was downregulated in B-PATPHs. Some genes involved in immune-related inflammatory responses were found to be differentially expressed in the present study. Thus, the aforementioned results indicated that host immune response genes were activated in wild *E. przewalskii* horses infested with horse botflies, which can help understand the immune status of the host.

**Figure 4:**
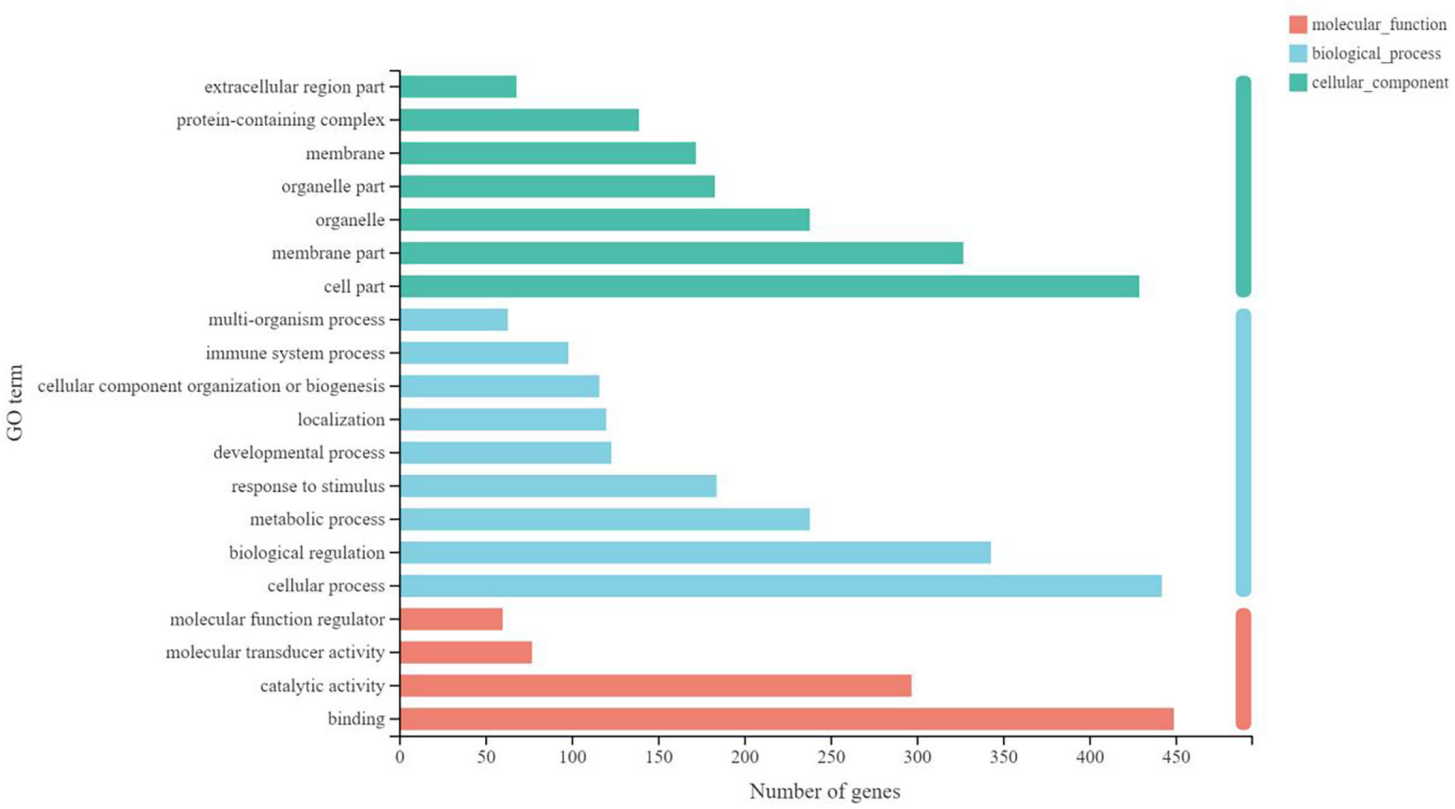
Gene function annotation in GO.

Finally, the functional enrichment analysis was performed to identify the functional differences in the DEGs between B-PATPHs and B-FATPHs. The GO annotation for unigenes suggested that these genes could be classified into three GO terms: biological process, cellular component, and molecular function (Figure 4). The GO functional enrichment analysis indicated that the most significantly different GO term was signaling pattern recognition receptor activity, while the defense response function contained the largest number of unigenes (64) (Supplementary Figure S6). This result is consistent with the findings of another study, where the defense response function is abundant in monkeys after immunization (79).

**Figure 5:**
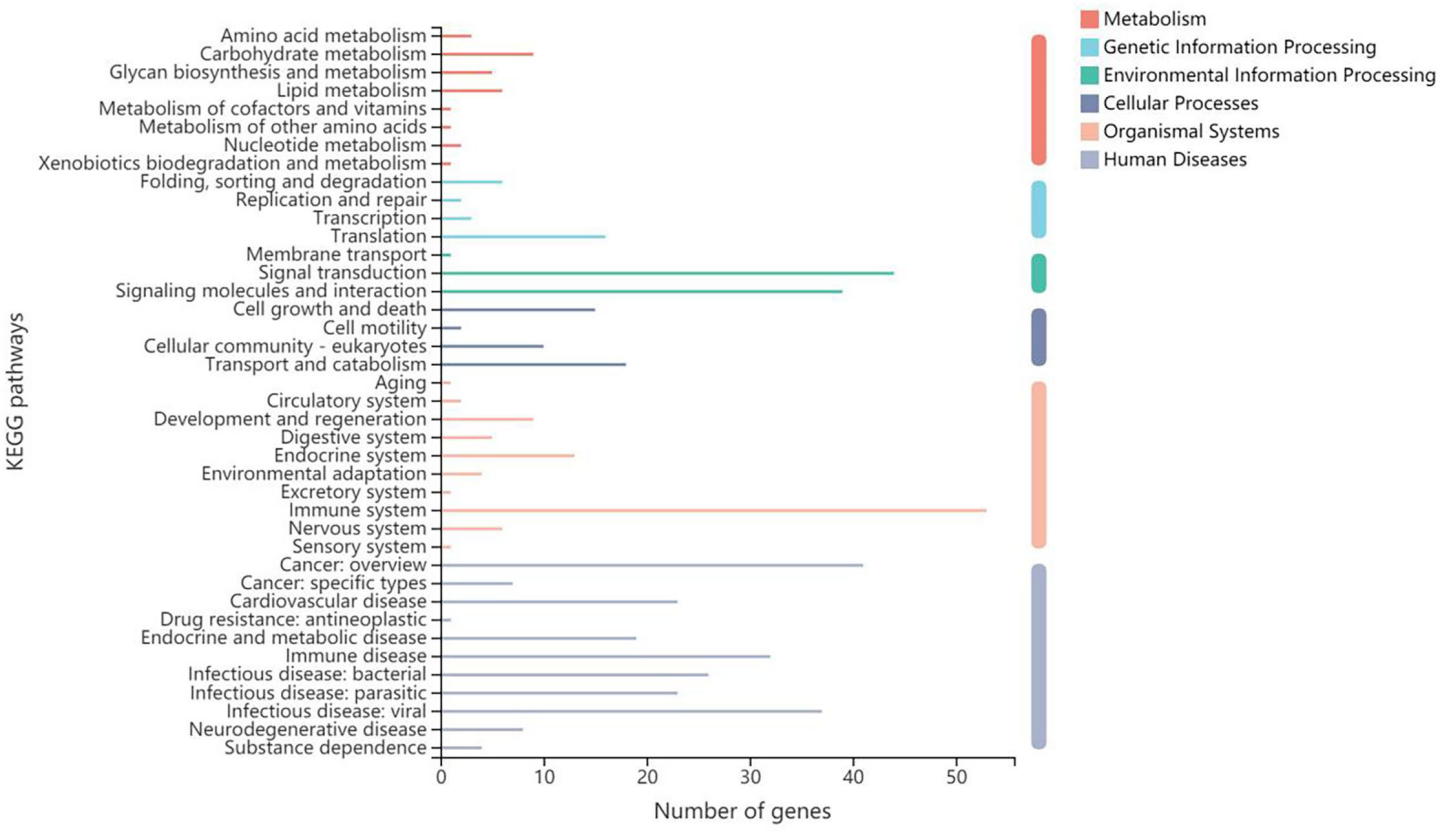
Gene function annotation in KEGG.

As for the participating KEGG functions, the first five annotated categories included human diseases, organismal systems, metabolism, cellular processes, genetic information processing, and environmental information processing (Figure 5). The top three second categories included the immune system, signal transduction, and cancer: overview. The KEGG enrichment analysis determined that the second-category DEGs were mainly enriched in pathways relating to cancer (47 unigenes), Th17 cell differentiation (36 unigenes), and osteoclast differentiation (Supplementary Figure S7); the latter two of were significantly different between B-PATPHs and B-FATPHs. Th17 is found to be involved in the immune response against parasite infection. While Ortiz Wilczyñski et al. (80) show that Th17 cell numbers are reduced in patients infected with helminths, in the mouse model, serum Th17 levels increase after infection with helminths (81). Thus, we assume that horse botfly infestation would trigger an immune response in *E. przewalskii* that might affect the progression of parasite infections with botflies.

### Associations Between Metabolites and Gene Expression

**Figure 6:**
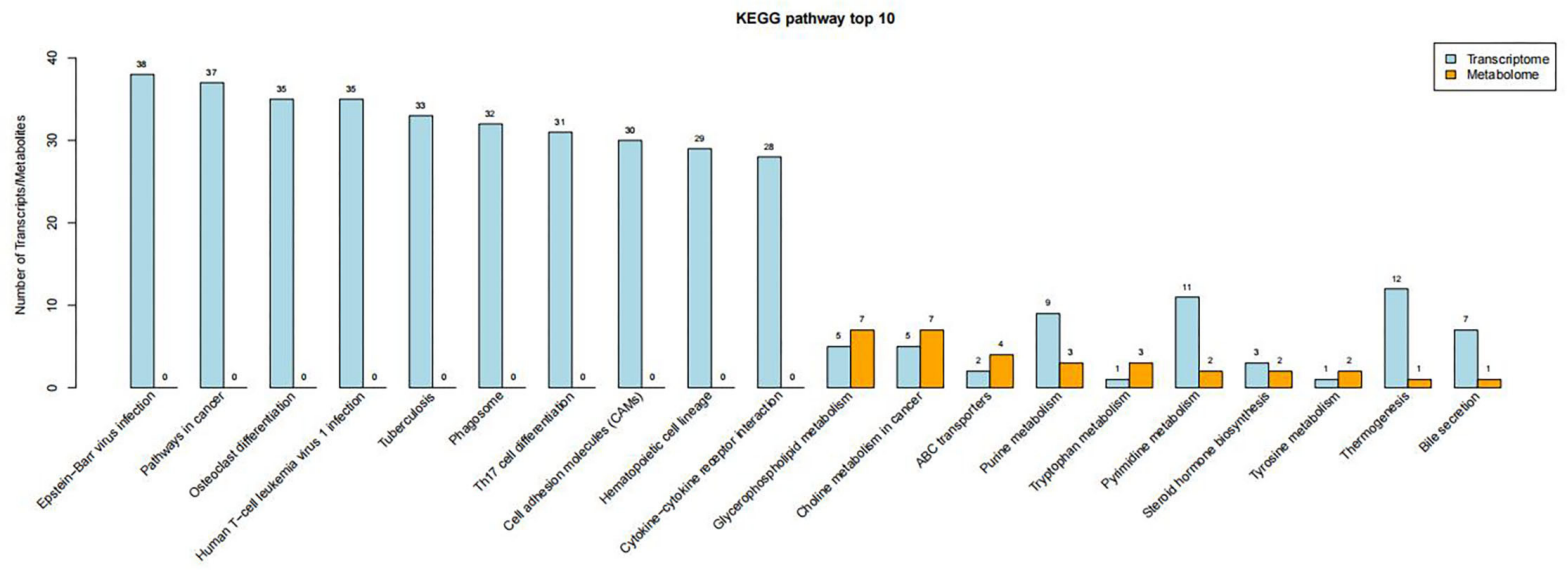
Top 10 KEGG pathways containing the most DEGs and altered metabolites.

**Figure 7:**
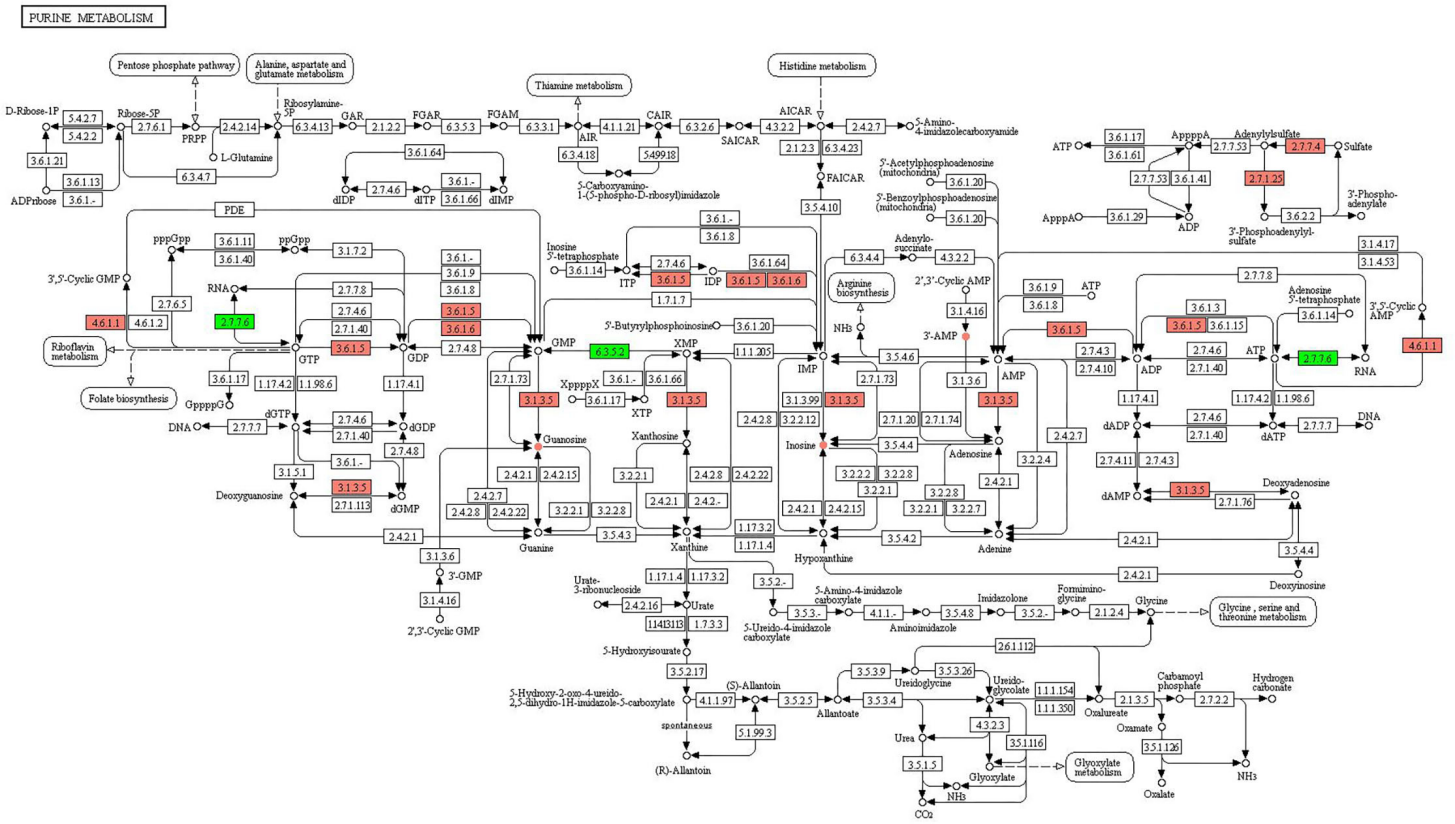
KEGG pathway of purine metabolism. The boxes represent gene products, and the circles represent metabolites. The gene products in red and green are the upregulated and downregulated genes, respectively. The metabolites in red and green are the upregulated and downregulated metabolites, respectively.

**Figure 8:**
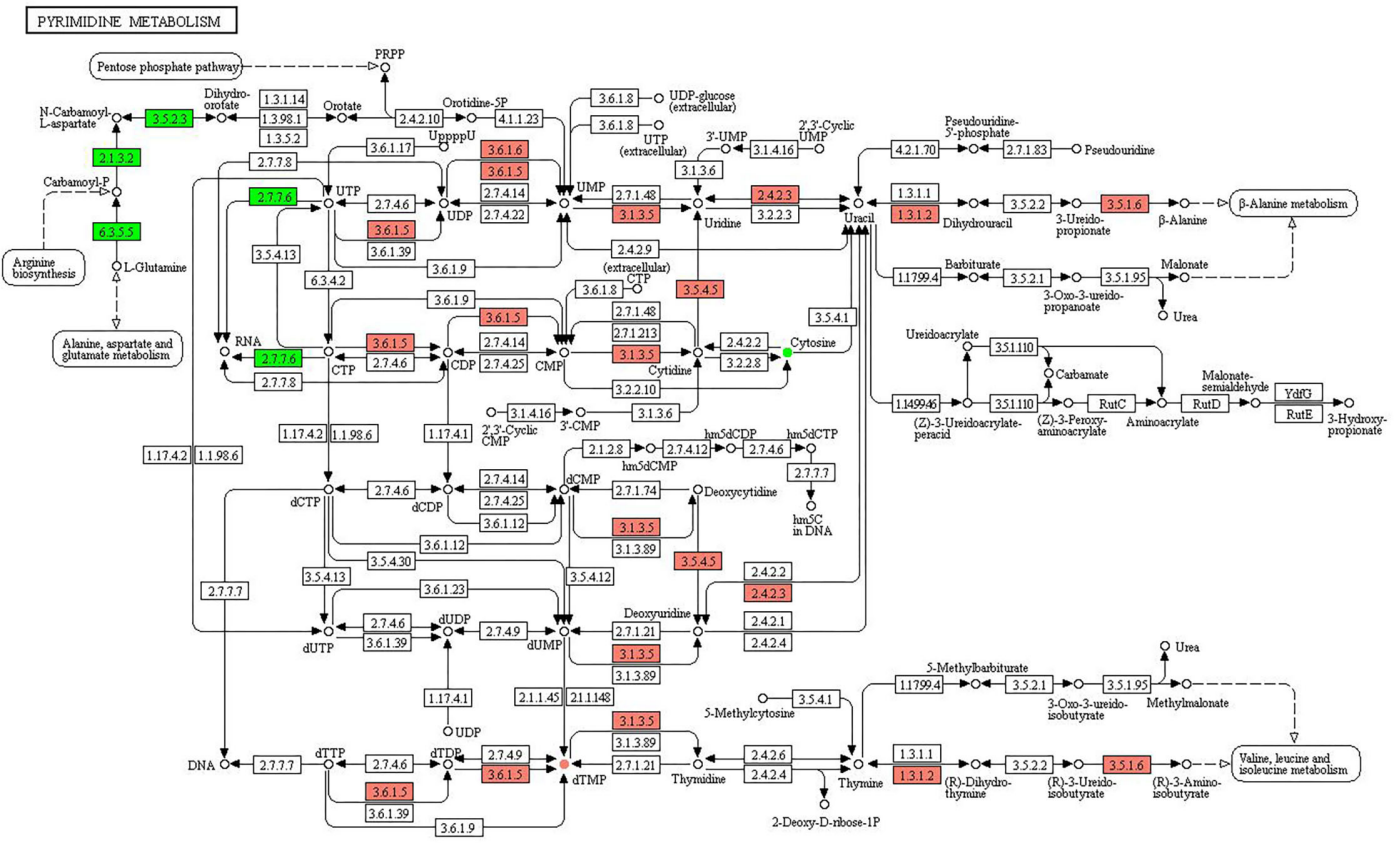
KEGG pathway of pyrimidine metabolism. The boxes represent gene products, and the circles represent metabolites. The gene products in red and green are the upregulated and downregulated genes, respectively. The metabolites in red and green are the upregulated and downregulated metabolites, respectively.

The altered metabolites and DEGs were commonly involved in 19 KEGG pathways, of which the top 10 were glycerophospholipid metabolism, choline metabolism in cancer, ABC transporters, purine metabolism, tryptophan metabolism, pyrimidine metabolism, steroid hormone biosynthesis, tyrosine metabolism, thermogenesis, and bile secretion (Figure 6). Among them, purine metabolism and pyrimidine metabolism contained the most altered metabolites and DEGs (Figures 7, 8, Supplementary Figures S8–S15).

In purine metabolism (Figure 7), the upregulated genes were involved in the processes of converting GTP to 3′,5′-cyclic GMP (4.6.1.1), GTP to GDP (3.6.1.5), dGMP to deoxyguanosine (3.1.3.5), GDP to GMP (3.6.1.5, 3.6.1.6), GMP to guanosine (3.1.3.5), IDP to ITP (3.6.1.5), IDP to IMP (3.6.1.5, 3.6.1.6), IMP to inosine (3.1.3.5), AMP to adenosine (3.1.3.5), ADP to AMP (3.6.1.5), ATP to ADP (3.6.1.5), ATP to 3′,5′-cyclic AMP (4.6.1.1), sulfate to adenylylsulfate (2.7.7.4), and adenylylsulfate to 3′-phosphoadenylyl-sulfate (2.7.1.25). The downregulated genes were involved in the process of GTP to RNA (2.7.7.6), XMP to GMP (6.3.5.2), and ATP to RNA (2.7.7.6). The upregulated metabolites were guanosine, inosine, and 3′ AMP, and no downregulated metabolites were identified.

Figure 8 shows the KEGG pathway of purine metabolism. The upregulated genes were associated with the pathways of UTP to UDP (3.6.1.5), UDP to UMP (3.6.1.5, 3.6.1.6), UMP to uridine (3.1.3.5), uridine to uracil (2.4.2.3), uracil to dihydrouracil (1.3.1.2), uracil to deoxyuridine (2.4.2.3), 3-ureido-propionate to β-alanine (3.5.1.6), CTP to CDP (3.6.1.5), CDP to CMP (3.6.1.5), CMP to cytidine (3.1.3.5), cytidine to uridine (3.5.4.5), dCMP to deoxycytidine (3.1.3.5), deoxycytidine to deoxyuridine (3.5.4.5), dUMP to deoxyuridine (3.1.3.5), dTTP to dTDP (3.6.1.5), dTDP to dTMP (3.6.1.5), dTMP to thymidine (3.1.3.5), thymine to (R)-dihydrothymine (1.3.1.2), and (R)-3-ureidoisobutyrate to (R)-3-aminoisobutyrate (3.5.1.6). The downregulated genes were involved in L-glutamine to carbamoyl-P (6.3.5.5), carbamoyl-P to *N*-carbamoyl-l-aspartate (2.1.3.2), *N*-carbamoyl-l-aspartate to dihydroorotate (3.5.2.3), CTP to RNA (2.7.7.6), and UTP to RNA (2.7.7.6). The upregulated and downregulated metabolites were dTMP and cytosine, respectively.

In mammals, when mitochondrial activity is unable to support energy demand, muscles produce ATP through the adenylate kinase reaction in the purine metabolism. The product of purine metabolism is uric acid (82). There are differences in purine metabolism in different species, which cause gout and renal stones in humans, but the effects of which on horses are not known (83). One study shows that uric acid levels increase when horses engage in strenuous activities (84). In contrast to purine metabolism, the pyrimidine metabolism shows little difference in different species. Pyrimidines are the critical nutrients and play an important role in maintaining the physiological homeostasis of the host, and the metabolic aberrations of pyrimidines disrupt nervous, mitochondrial, and hematological systems (85). This led us to assume that horse botfly infestation may increase the energy consumption, affect the nutrient absorption, and damage normal physiological processes of the horses.

## Conclusion

The parasite infection is well known to influence the intestinal microbial community and composition. This study aims to explain the whole process after the host was infected with parasites and uncover a major role of intestinal microbiota and its metabolites in the modulation of the host immune response. The results show that while changing intestinal microbiota, parasites also adjust the types and levels of metabolites microbiota produce. Then, the altered microbiota and their metabolites will stimulate the immune response of the host, which might occur through purine and pyrimidine metabolism. This study can provide some reference information for further studies, such as fecal microbiota transplant to restore the host immunity and develop probiotics and microbial-related drugs. Future studies are needed and valuable.

## Data Availability Statement

The datasets presented in this study can be found in online repositories. The names of the repository/repositories and accession number(s) can be found in the article/supplementary material.

## Ethics Statement

The animal study was reviewed and approved by Ethics Committee of the Beijing Forestry University (EAWC_BJFU_2020012).

## Author Contributions

DH, CW, YQ, ME, XL, and KL conceived the experiments and undertook sampling work. DH, DZ, KL, and HC analyzed the results and wrote the manuscript. All authors read and approved the final manuscript.

## Funding

This research was funded by the China Postdoctoral Science Foundation with (No. 2020TQ0047); the National Science Foundation of China (No. 31670538); the Species Project (2018) of Department for Wildlife and Forest Plants Protection, NFGA of China; and the Forestry Fund of LiBin (02210823).

## Conflict of Interest

The authors declare that the research was conducted in the absence of any commercial or financial relationships that could be construed as a potential conflict of interest.

## Publisher's Note

All claims expressed in this article are solely those of the authors and do not necessarily represent those of their affiliated organizations, or those of the publisher, the editors and the reviewers. Any product that may be evaluated in this article, or claim that may be made by its manufacturer, is not guaranteed or endorsed by the publisher.
