# Supplementary figures and images for: The Role of Intestinal Microbial Metabolites in the Immunity of Equine Animals Infected With Horse Botflies
Source: Front Vet Sci. 2022 Jun 22;9:832062. doi: 10.3389/fvets.2022.832062 (PMC9257286; doi:10.3389/fvets.2022.832062)

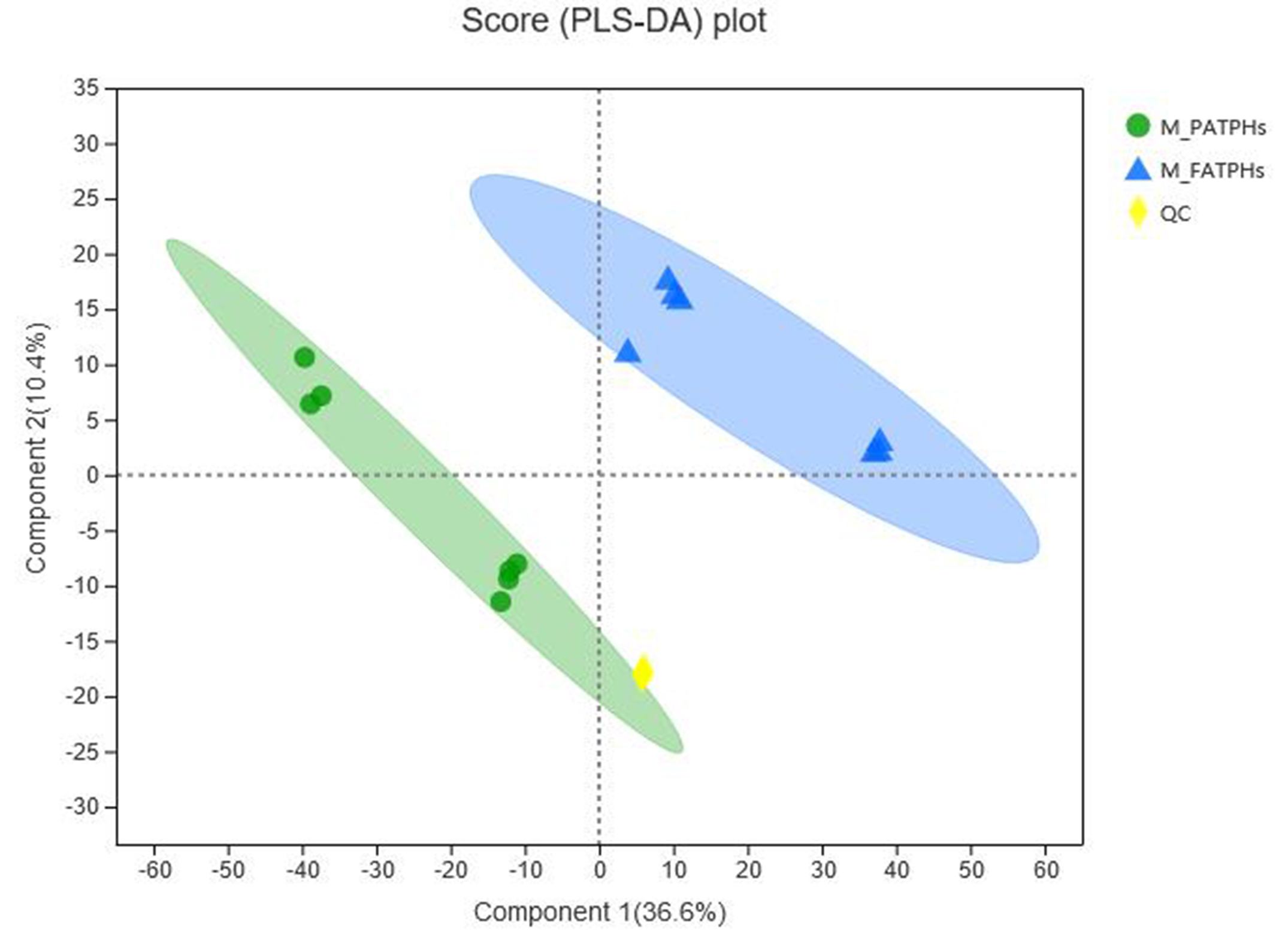

Supplement: Supplementary file 1 [file Image_1.JPEG]

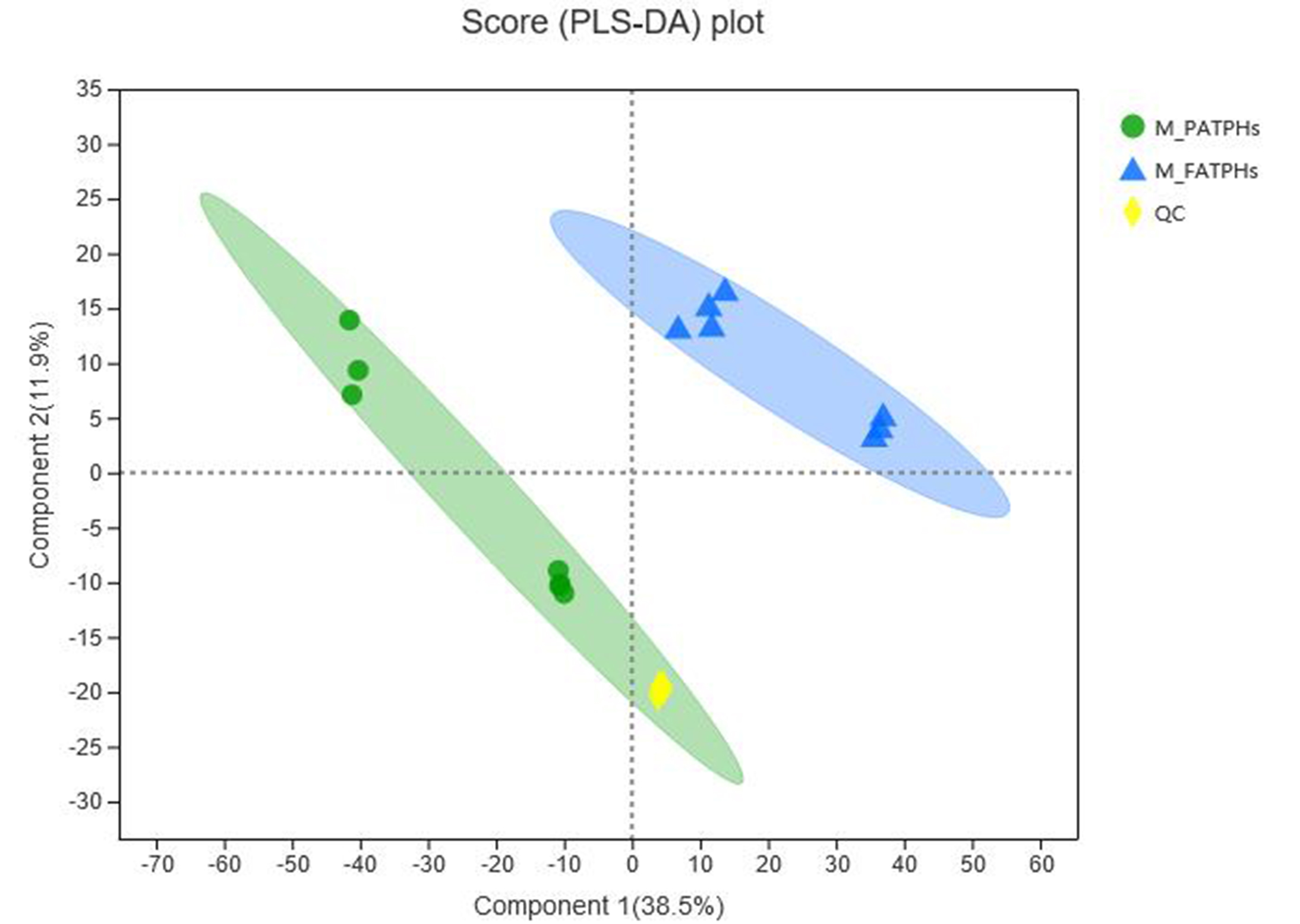

Supplement: Supplementary file 2 [file Image_2.JPEG]

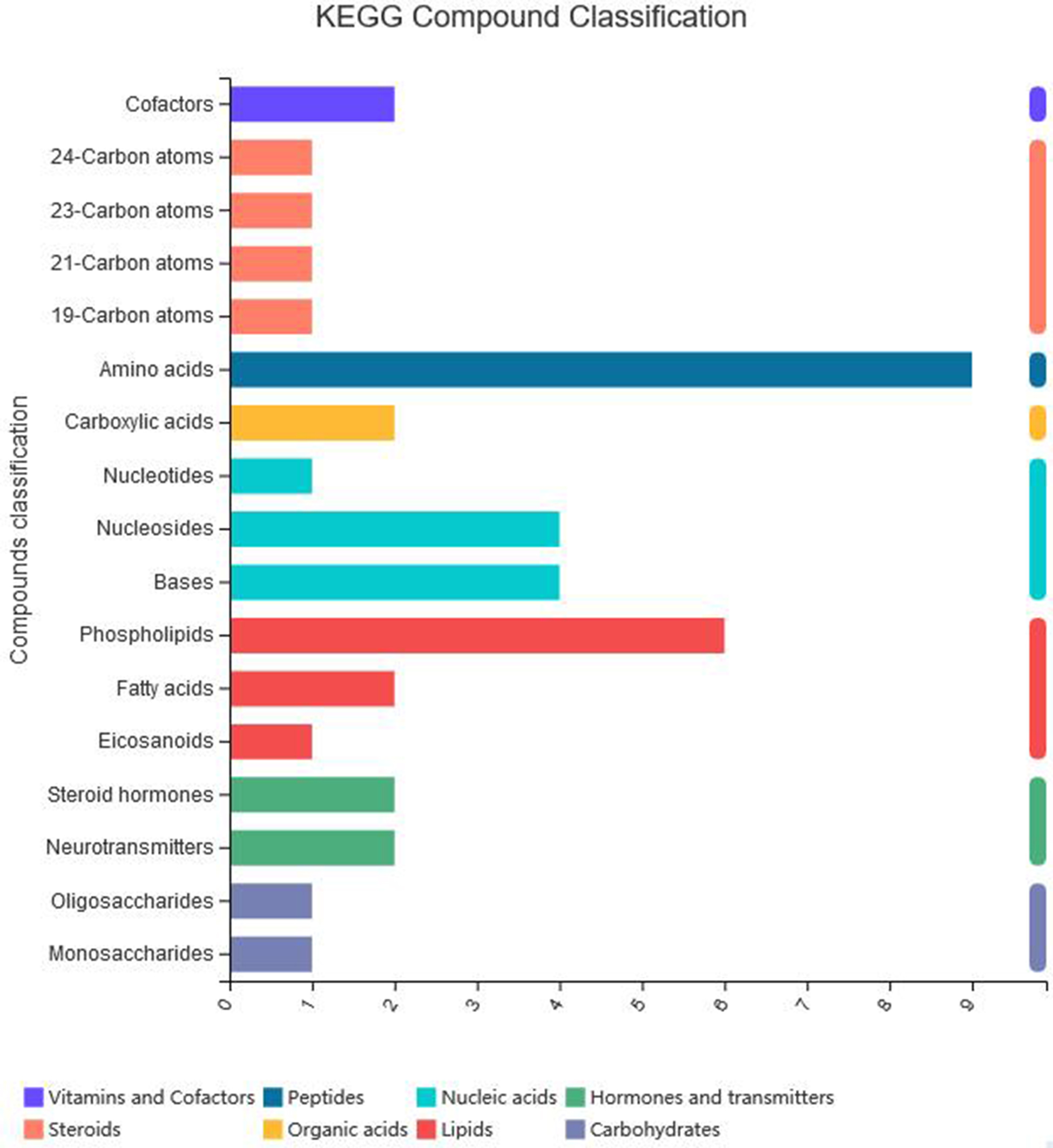

Supplement: Supplementary file 3 [file Image_3.JPEG]

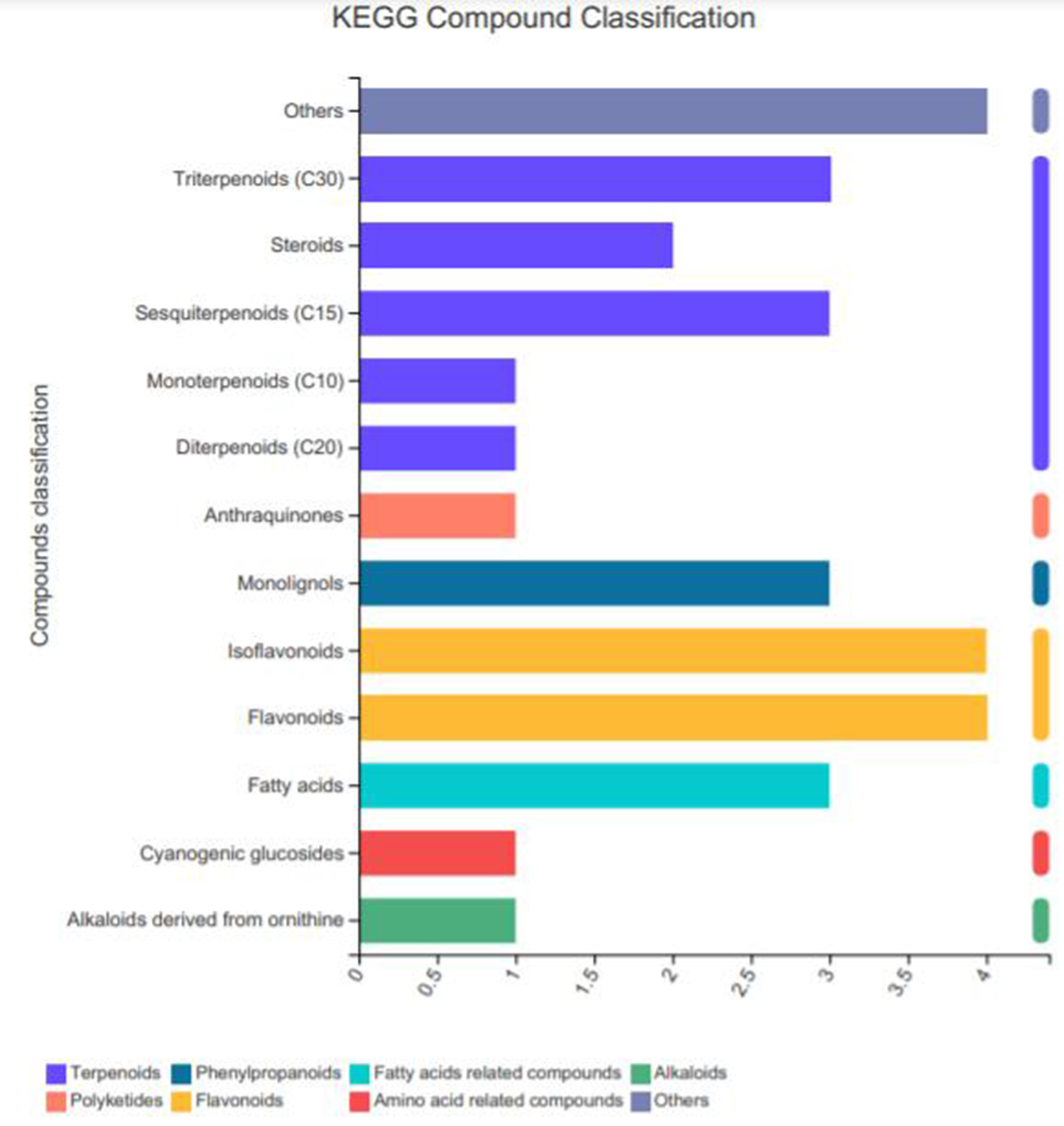

Supplement: Supplementary file 4 [file Image_4.JPEG]

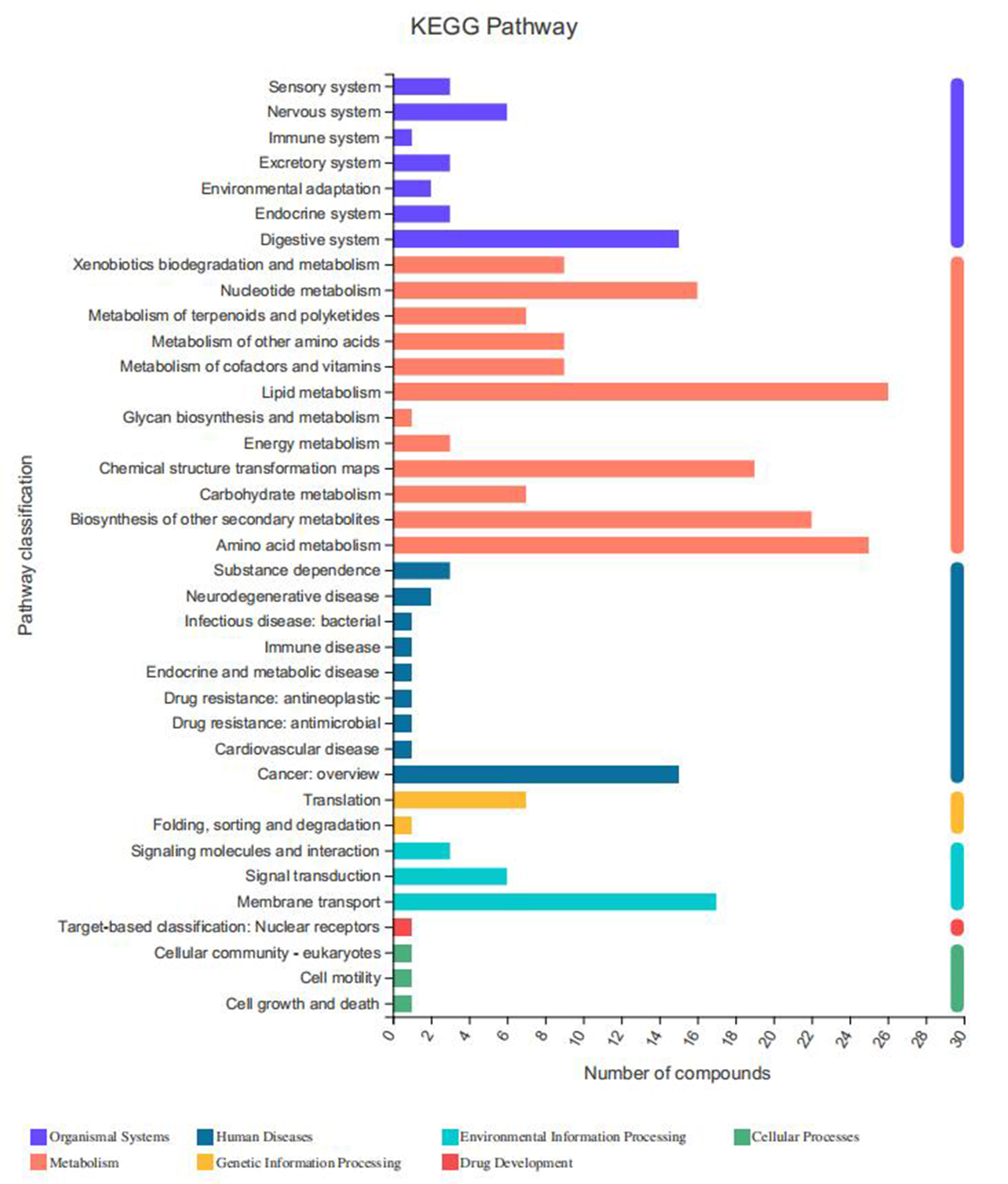

Supplement: Supplementary file 5 [file Image_5.JPEG]

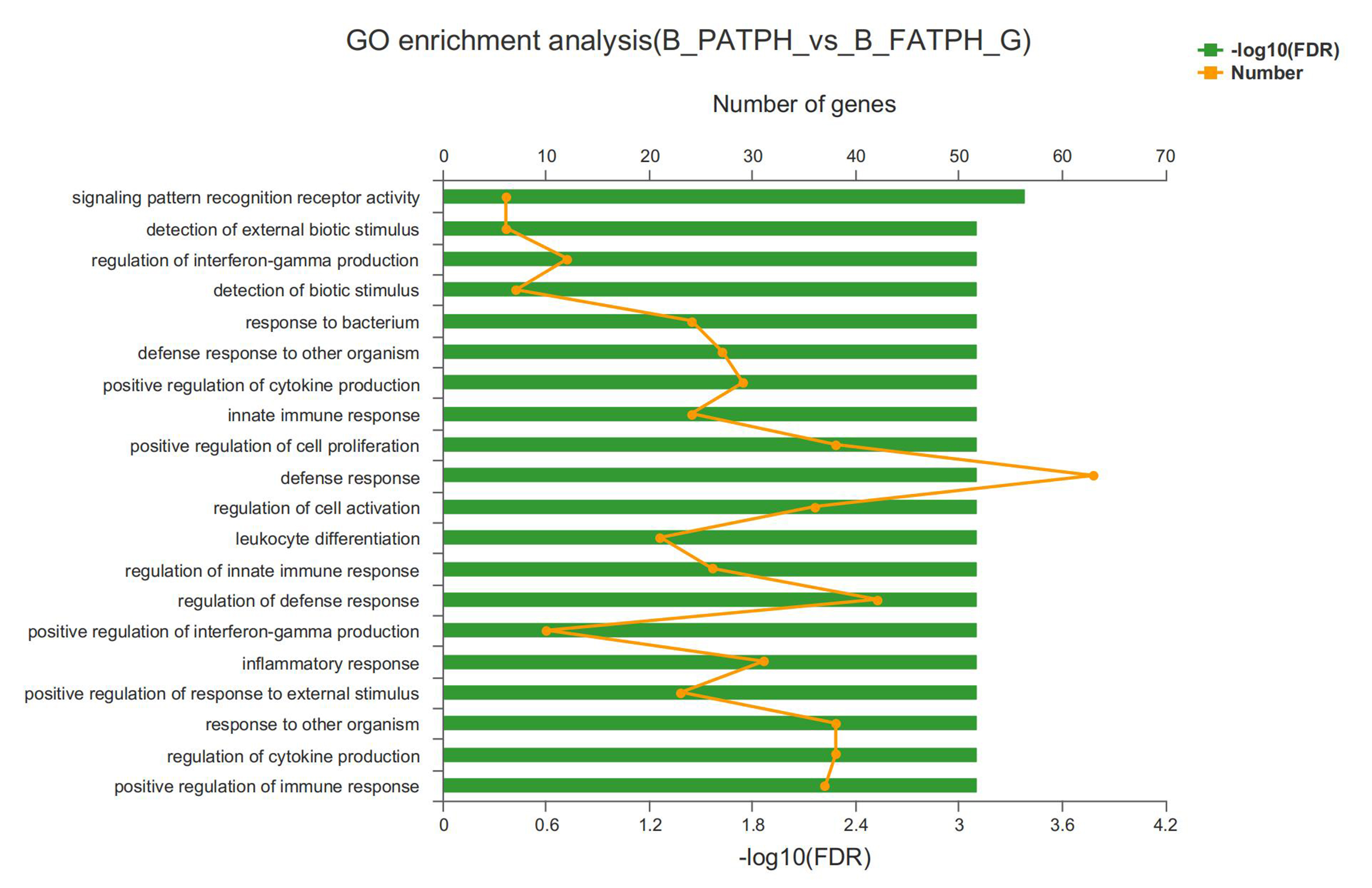

Supplement: Supplementary file 6 [file Image_6.JPEG]

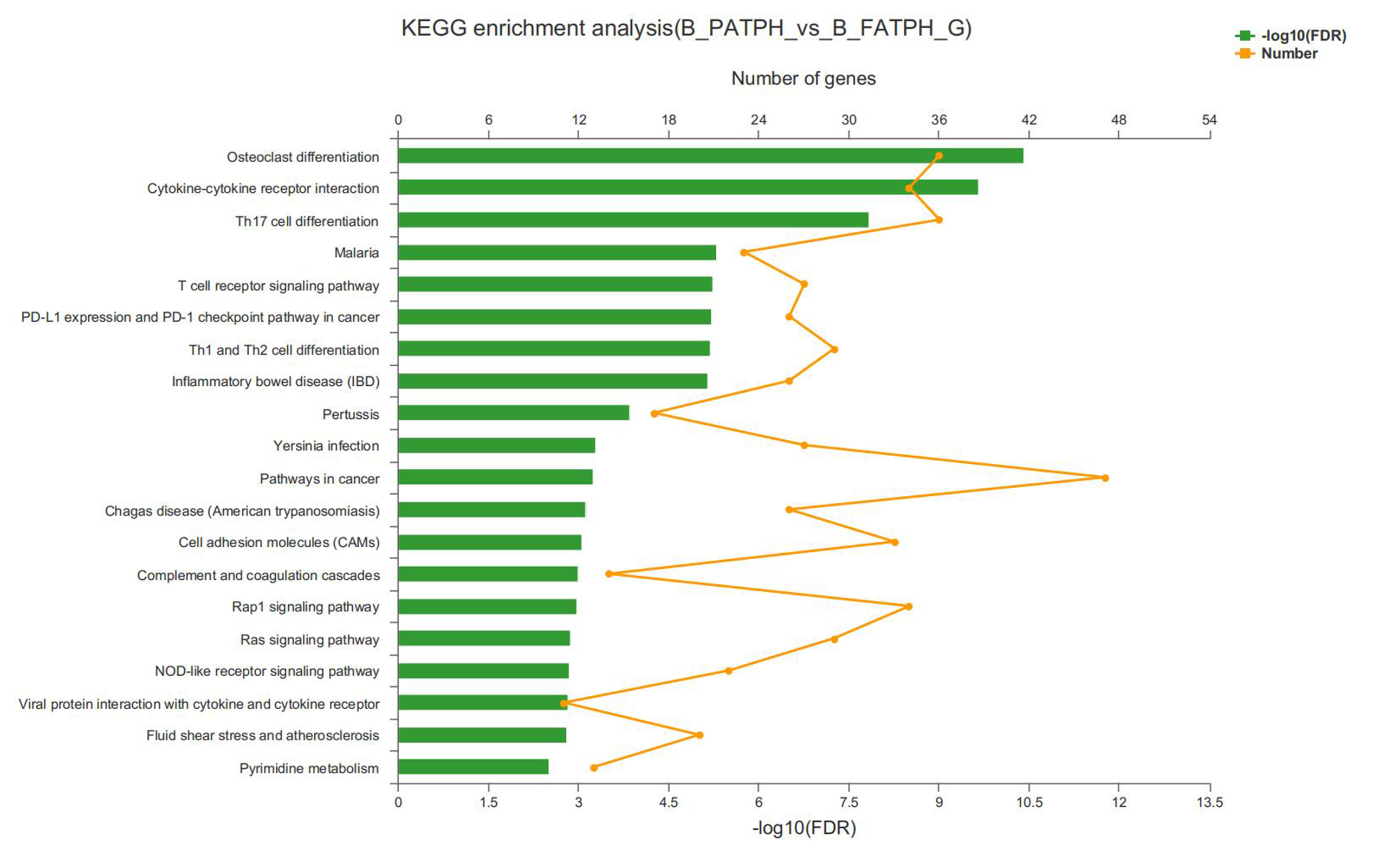

Supplement: Supplementary file 7 [file Image_7.JPEG]

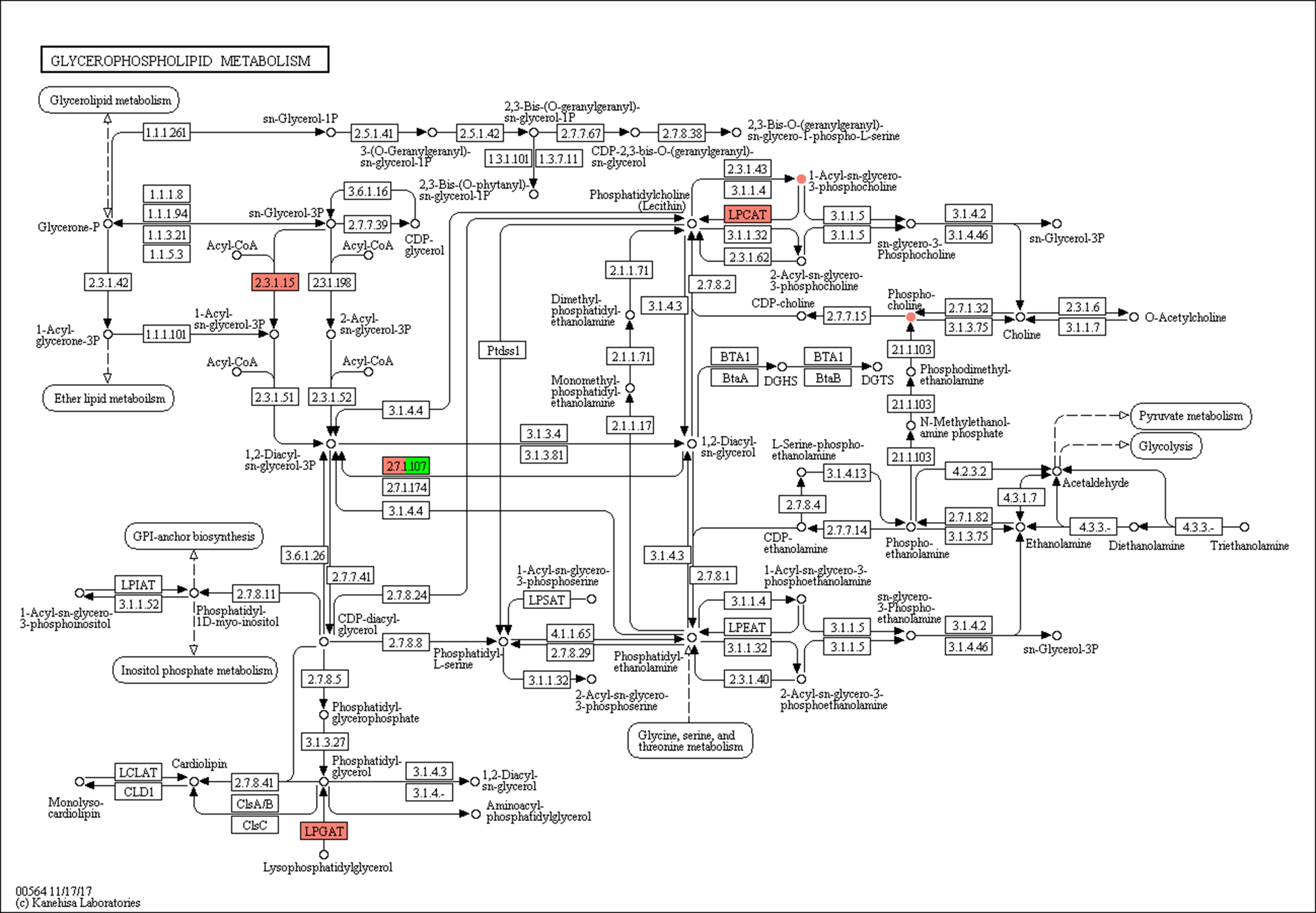

Supplement: Supplementary file 8 [file Image_8.PNG]

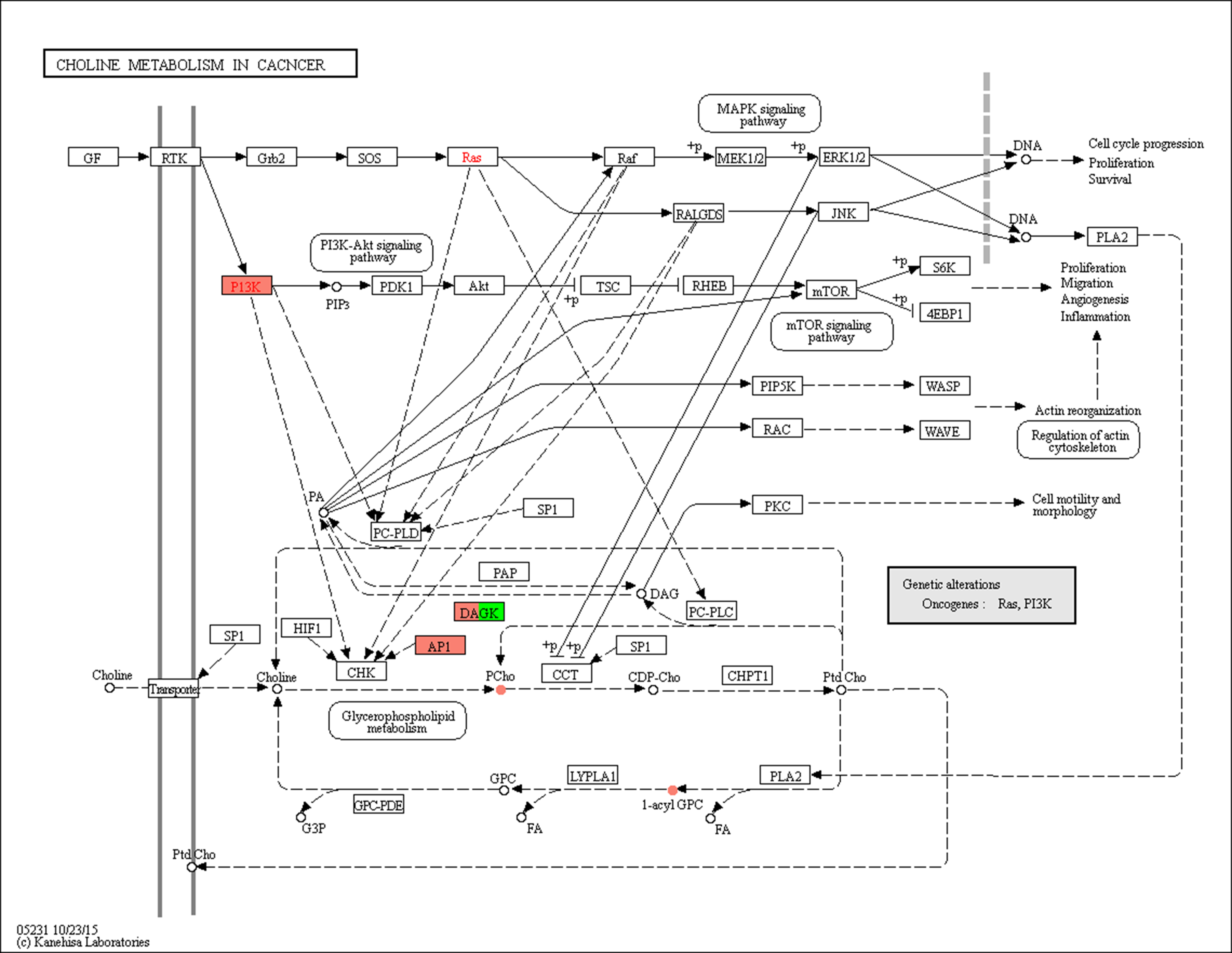

Supplement: Supplementary file 9 [file Image_9.PNG]

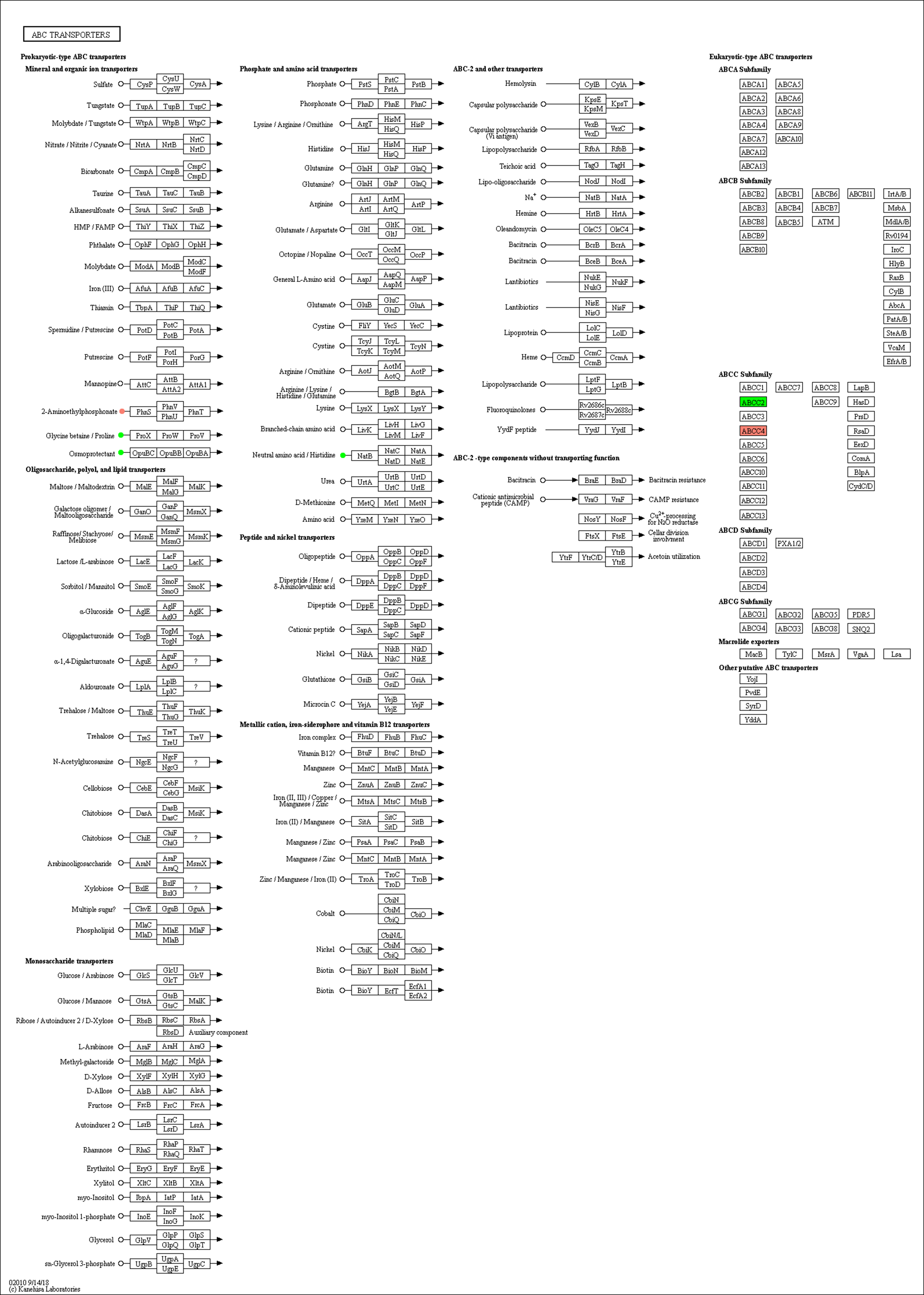

Supplement: Supplementary file 10 [file Image_10.PNG]

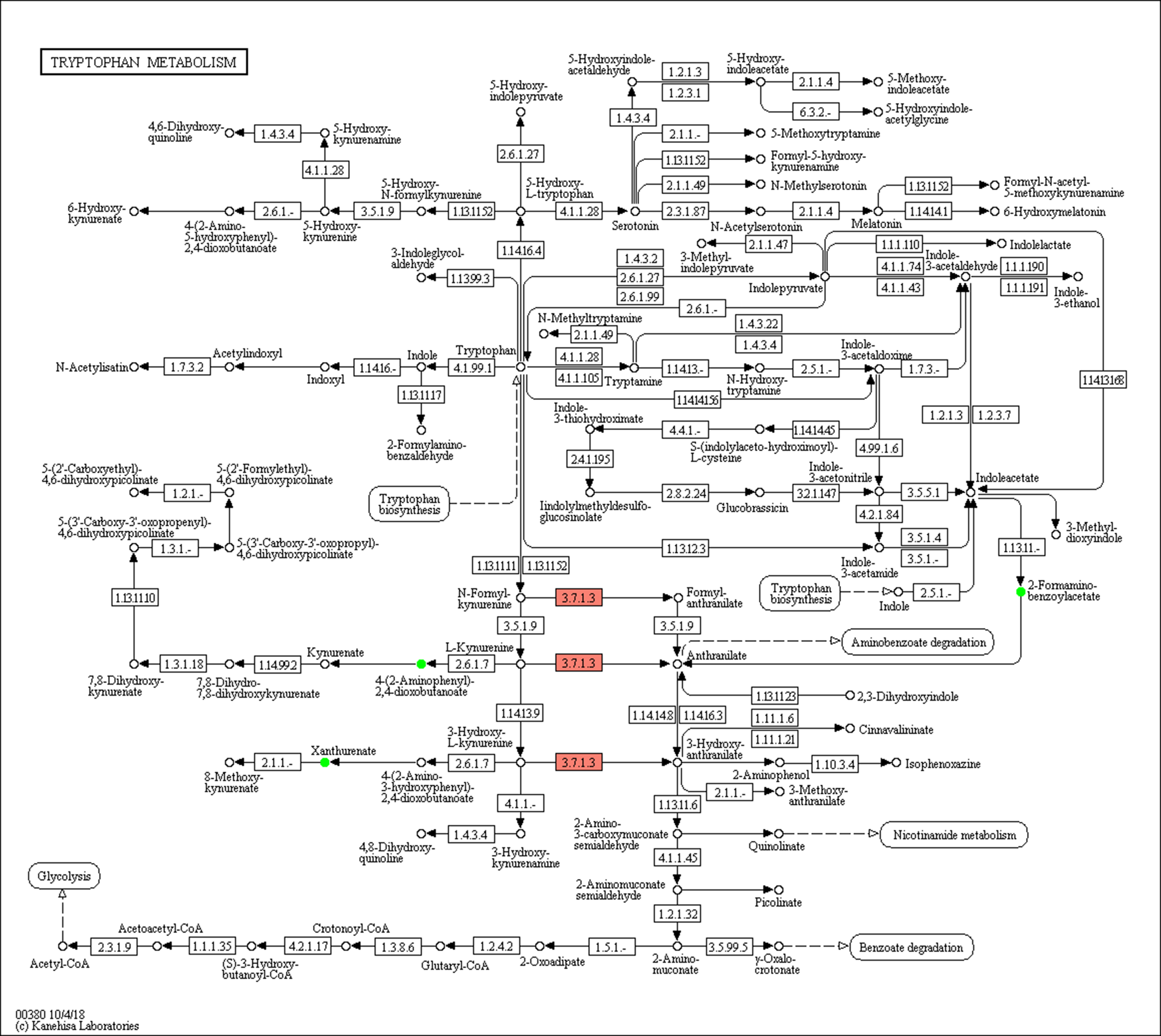

Supplement: Supplementary file 11 [file Image_11.PNG]

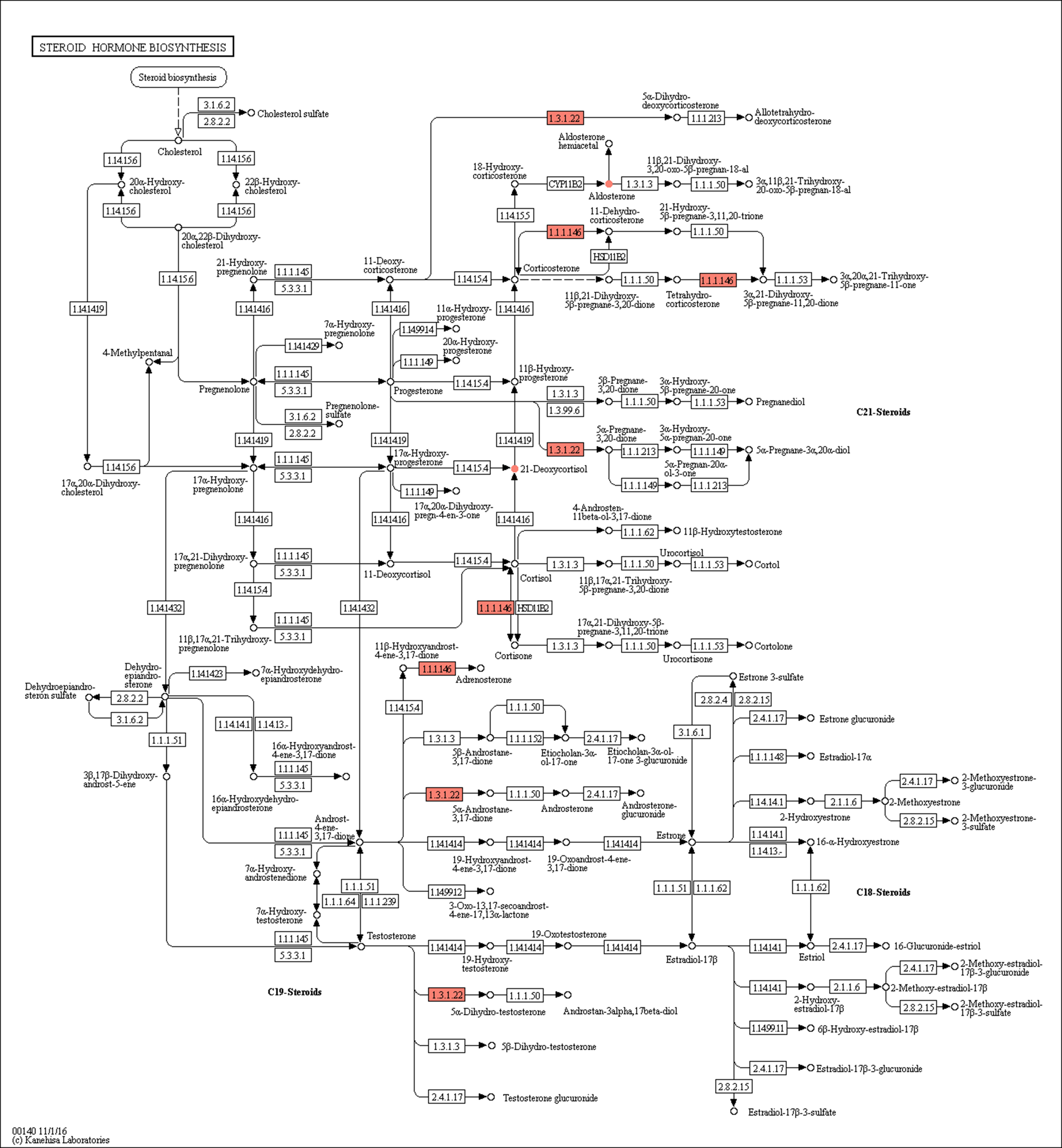

Supplement: Supplementary file 12 [file Image_12.PNG]

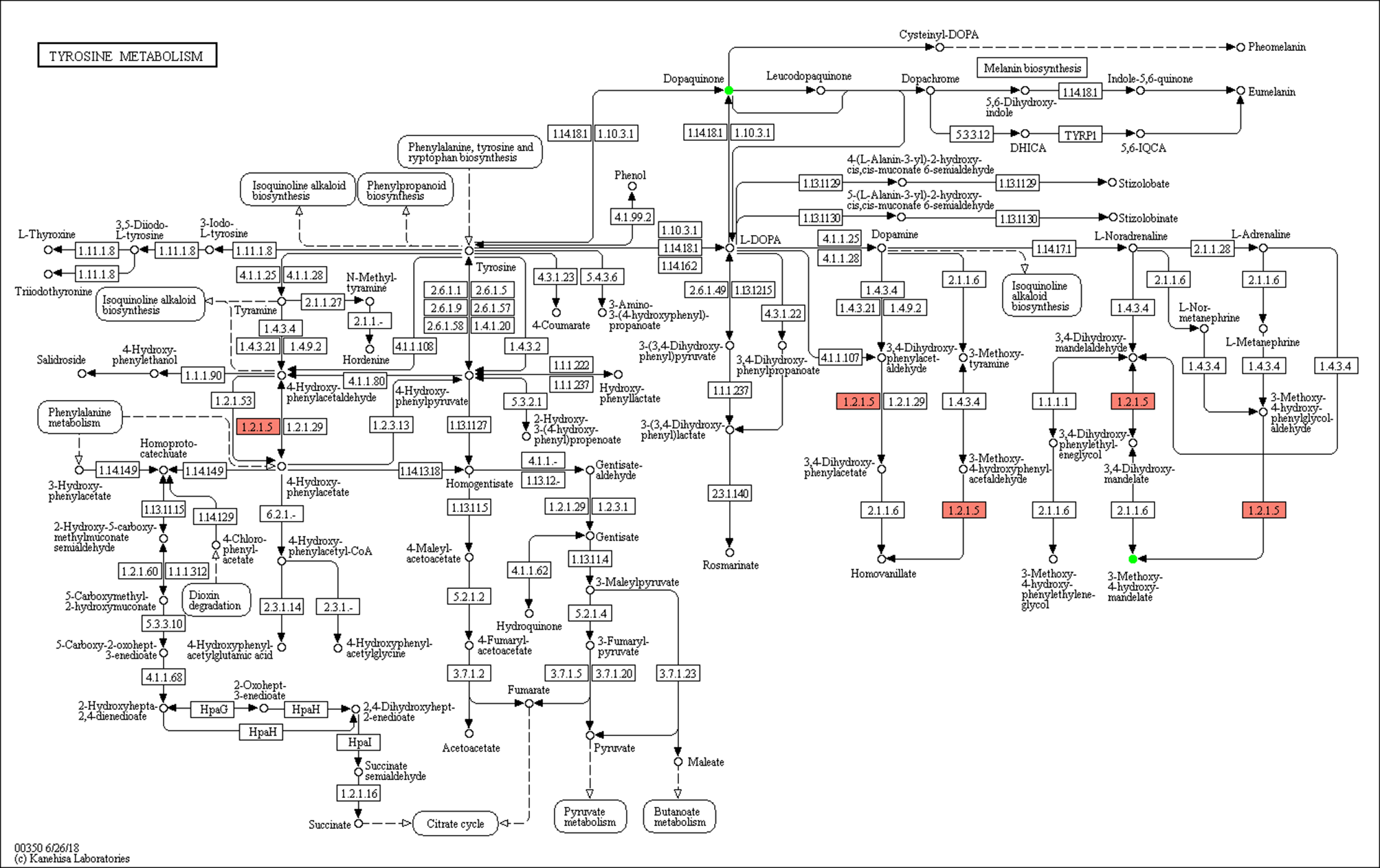

Supplement: Supplementary file 13 [file Image_13.PNG]

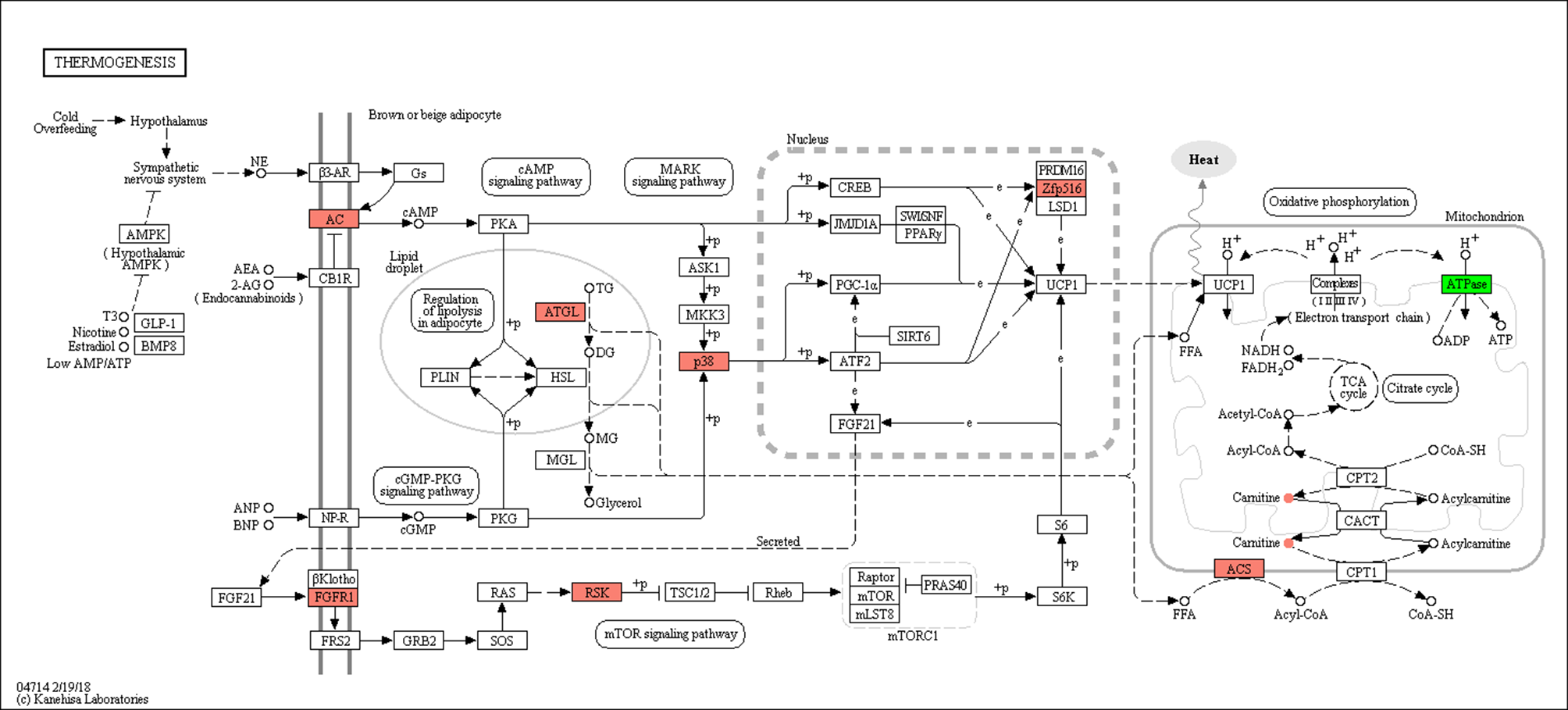

Supplement: Supplementary file 14 [file Image_14.PNG]

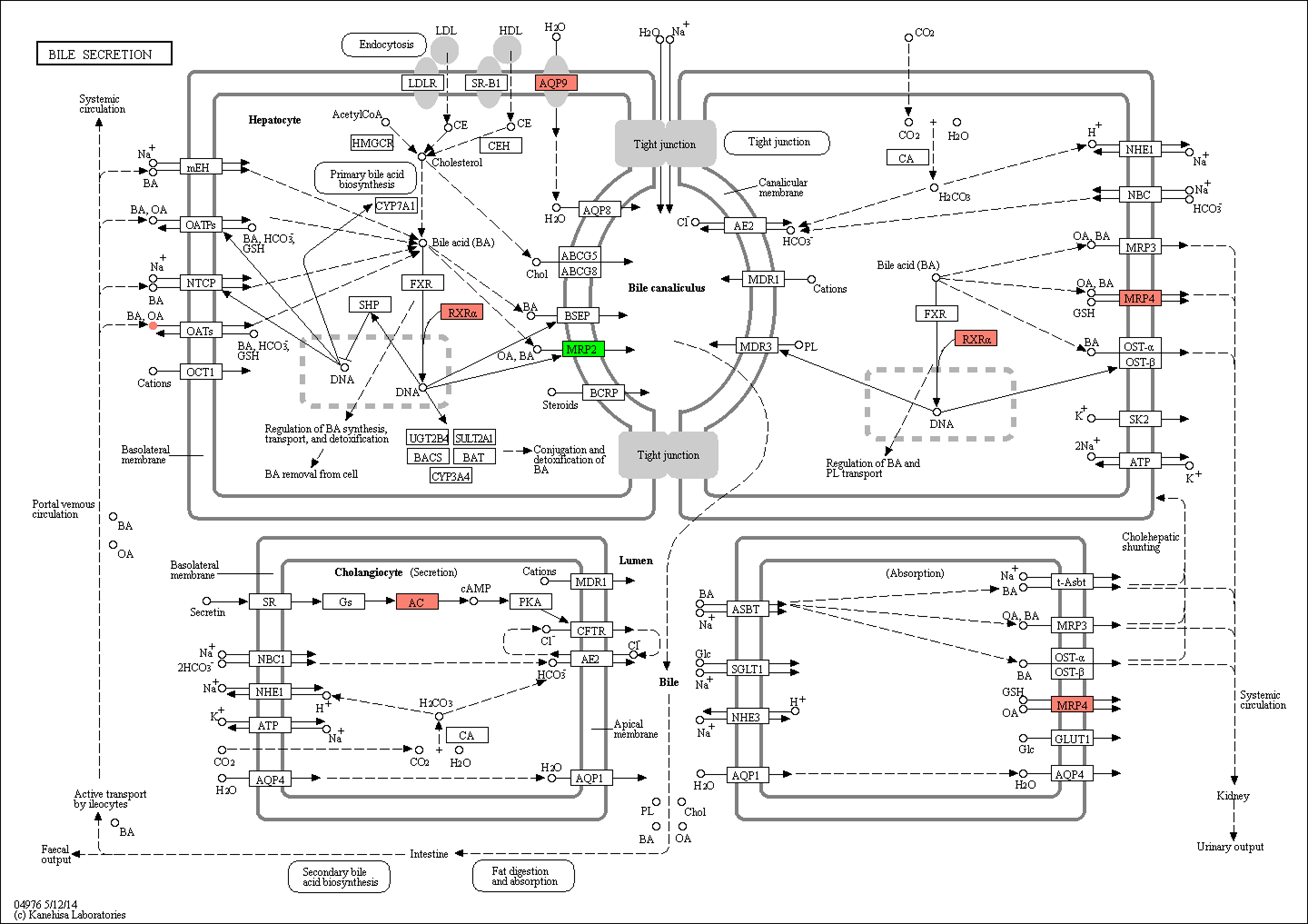

Supplement: Supplementary file 15 [file Image_15.PNG]
